# Supplementary material for: Cellular lensing and near infrared fluorescent nanosensor arrays to enable chemical efflux cytometry
Source: Nat Commun. 2021 May 25;12:3079. doi: 10.1038/s41467-021-23416-1 (PMC8149711; doi:10.1038/s41467-021-23416-1)
Supplement: Supplementary file 1 — Supplementary Information [file 41467_2021_23416_MOESM1_ESM.pdf]

Supporting Information for

Cellular Lensing and Near Infrared Fluorescent Nanosensor Arrays to Enable Chemical Efflux Cytometry

Soo-Yeon Cho,[1] *Xun Gong,*[1] *Volodymyr B. Koman,*[1] *Matthias Kuehne,*[1] *Sun Jin Moon,*[1] *Manki Son,*[1] *Tedrick Thomas Salim Lew,*[1,2] *Pavlo Gordiichuk,*[1] *Xiaojia Jin,*[1] *Hadley D. Sikes,*[1] *Michael S. Strano*[1,*]

[1] Department of Chemical Engineering, Massachusetts Institute of Technology, Cambridge, MA 02139, United States

[2] Institute of Materials Research and Engineering (IMRE), Agency for Science, Technology and Research (A*STAR), Singapore 138634

*Corresponding author: strano@mit.edu

Table of contents:

1. Nanosensor Characterization.

Supplementary Figure 1. Photograph and UV-vis-nIR absorption spectra of SWNT/$(GT)_{15}$ nanosensor dispersions

2. Characterizations of nanosensor integrated microfluidics (NIM).

Supplementary Table 1. Specifications of commercial microfluidic channel
Supplementary Figure 2. nIR mapping of NIM (a) with and (b) without ATPES treament for EISA
Supplementary Figure 3. Uniformity of nanosensor array integrated with microfluidic channel
Supplementary Figure 4. AFM characterizations of nanosensor array of microfluidic channel
Supplementary Figure 5. Nanosensor concentration effects on ESIA process

3. Observation and characterization of nIR cellular lensing effect.

Supplementary Figure 6. Optical microscope images of human monocytes for experiments
Supplementary Figure 7. Effects of underlying nanosensor array on cellular lensing
Supplementary Figure 8. Effects of excitation laser power control on cellular lensing
Supplementary Figure 9. Reliable nIR lensing profiles of single cell type
Supplementary Figure 10. Z-stage control experiments for investigating focal point formation of nIR lensing effect
Supplementary Figure 11. Human umbilical vein cells (HUVECs) monitoring using NIM

4. FDTD numerical modeling of photonic nanojet effect of cells.

Supplementary Figure 12. FDTD modeling results for nIR photonic nanojet effects with various cell size
Supplementary Figure 13. FDTD modeling results for nIR photonic nanojet effects with various cell eccentricity
Supplementary Figure 14. FDTD modeling results for nIR photonic nanojet effects with various cell RI
Supplementary Figure 15. FDTD modeling results for nIR photonic nanojet effects with various beam wavelength
Supplementary Figure 16. nIR lensing profiles of the cell with different focusing points

5. Chemical efflux monitoring using nIR lensing effect.

Supplementary Figure 17. Differentiations of monocyte (U937) into macrophage with PMA activations
Supplementary Figure 18. Real-time monitoring of nIR signal from multiple monocytes
Supplementary Figure 19. Monitoring of H_2O_2 efflux from stopped and moving cells
Supplementary Note 1. H_2O_2 efflux wave modeling
Supplementary Figure 20. H_2O_2 efflux wave modeling
Supplementary Figure 21. Automatic nIR image analysis program using MATLAB

6. 3D cytometry plots.

Supplementary Figure 22. 3D cytometry plots of -PMA and +PMA monocyte populations

7. 2D Kernel density estimations.

Supplementary Figure 23. 2D Kernel density estimations of cytometry plots in Figure 5c

Supplementary Figure 24. 2D Kernel density estimations of cytometry plots in Figure 5e

8. NCC data comparison with commercial H_2O_2 assay kit.

Supplementary Figure 25. Comparison of NCC and commercial assay

9. Throughput calculation.

Supplementary Figure 26. Comparison of NCC and commercial assay

10. Parallel channel approach.

Supplementary Figure 27. Multi-channel NIM approach for large number cell analyzing.

11. Cell viability assay.

Supplementary Figure 28. Viabilities of cells from incubators and after flowing channel

12. Versatility of NCC platform.

Supplementary Figure 29. iNOS (inducible nitric oxide synthase) heterogeneity monitoring of macrophage populations.

1. Nanosensor characterization.

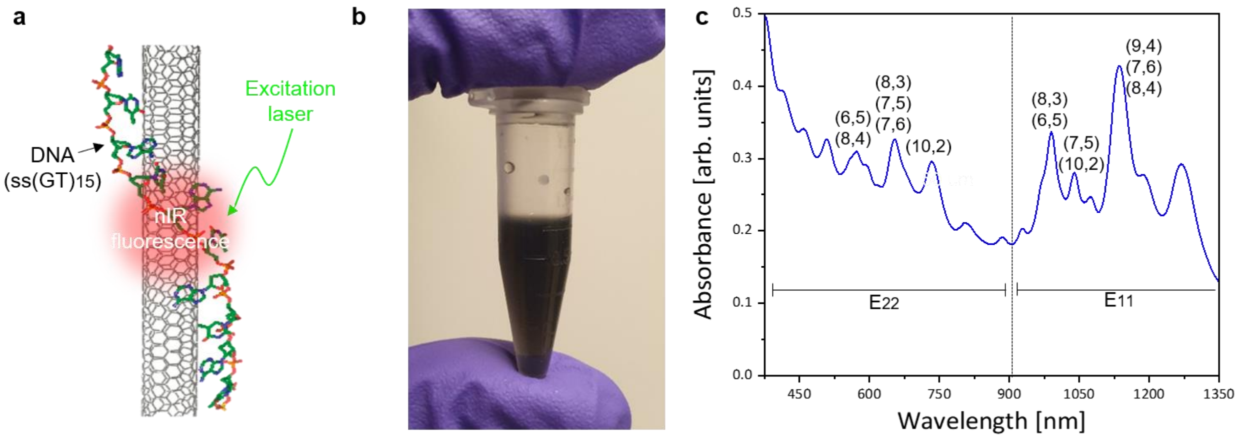

Supplementary Figure 1. (a) Schematic of single strand $(GT)_{15}$ oligonucleotide-wrapped SWNT (SWNT/$(GT)_{15}$). (b) Photograph of SWNT/$(GT)_{15}$ dispersions in 0.1 M NaCl solution. (c) UV-Vis-nIR absorption spectra of SWNT/$(GT)_{15}$ nanosensor dispersions. Nanosensor concentration in the dispersion was estimated using an extinction coefficient of $\mathcal{E}_{632\ nm}$ = 0.036 $(mg/L)^{-1}$. Final concentration of SWNT is 10 - 80 mg/L.

2. Characterizations of nanosensor integrated microfluidics (NIM).

**Outer dimensions (width X length)**	75.5 X 25.5 mm^2
**Adapters**	Female Luer
**Number of channels**	6
**Channel volume**	1.7 µL
**Channel height**	0.1 mm
**Channel length**	17 mm
**Channel width**	1 mm
**Volume per reservoir**	60 µL
**Growth area**	0.17 cm^2 per channel
**Coating area using 1.7 uL**	0.34 cm^2 per channel
**Bottom**	Ibidi Polymer Coverslip

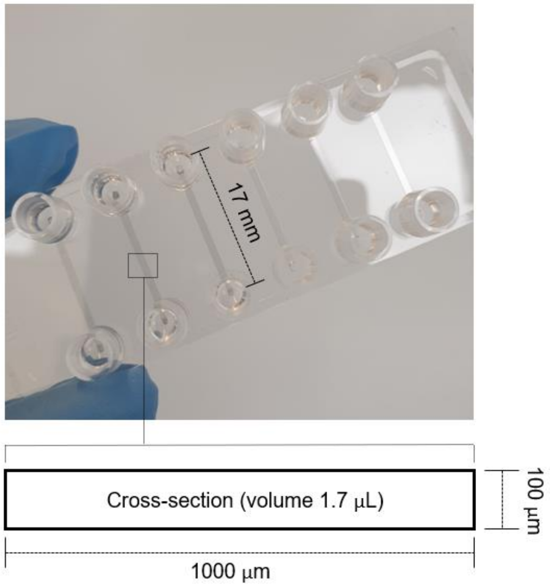

Supplementary Table 1. Specifications of commercial microfluidic channel (purchased from ibidi^{R} (µ-Slide VI 0.1, ibiTreat).

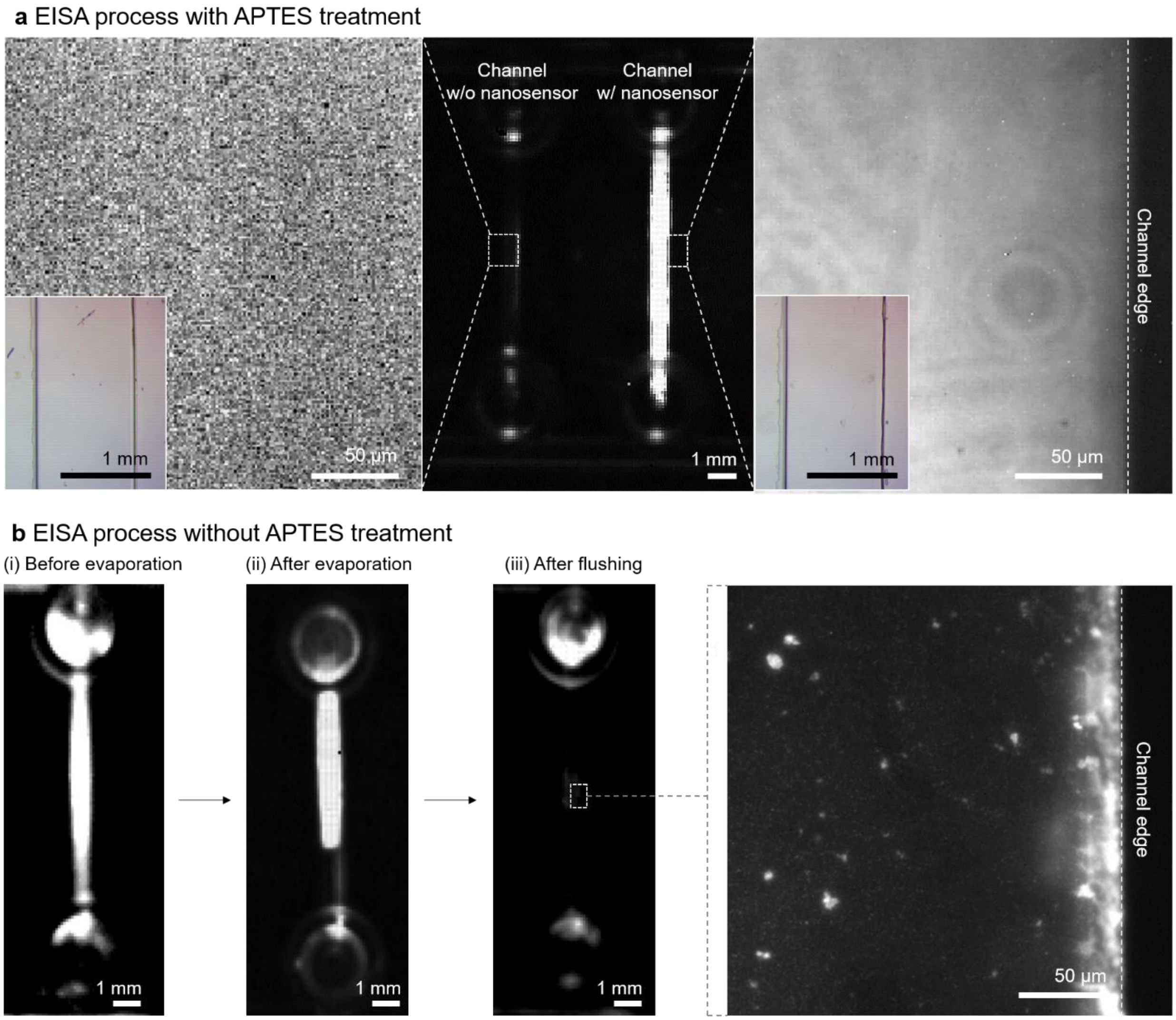

Supplementary Figure 2. nIR mapping of NIM (a) with and (b) without ATPES treament for EISA. Inset (bottom-left of (a)): optical miscroscope images of NIM.

Supplementary Figure 2a shows that NIM uniformly emits the significant nIR fluorescence throughout the entire channel inner surface, on the contrary, microfluidic channel without nanosensor could not be visualized at all due to lack of nIR signal. Optical microscope images (inset, bottom-left) show that NIM was transparent similar to pristine channels without nanosensors, indicating that SWNT/$(GT)_{15}$ nanosensors are reasonably uniformly integrated within microfluidic channel via EISA without any significant aggregations or defects. However, NIM without APTES treatment showed severe nanosensor aggregation during EISA process

(**Supplementary Figure 2b**). Consequently, nanosensors were significantly removed with PBS flushing, indicating that surface chemistry of the microfluidic channel is important to induce uniform and stable EISA process for NIM fabrication.

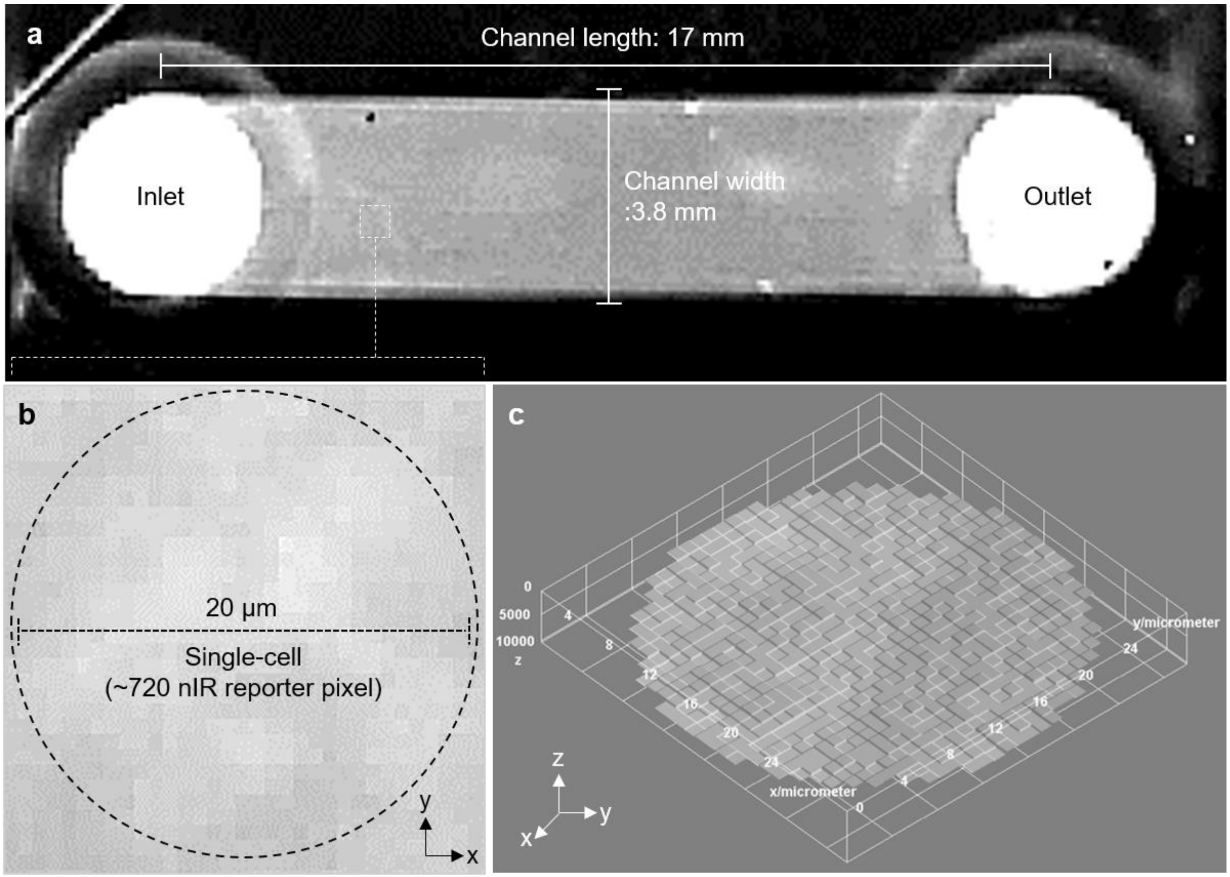

Supplementary Figure 3. Uniformity of nanosensor array integrated with microfluidic channel. (a) nIR image of 3.8 mm wide and 17 mm long microfluidic channel (μ-Slide VI 0.4, ibidi^R) with nanosensor integration. SWNT/(GT)$_{15}$ nanosensors were uniformly integrarted even with large-area channel srufaces (64.6 mm^2). (b) Magnified nIR image (single cell size; 20 μm) and (c) its 2D pixel intensity profile. SWNT/(GT)$_{15}$ nanosensors are homogeneously and densely assembled on whole area of the microfluidic channel with 720 nIR reporter pixels for a single cell.

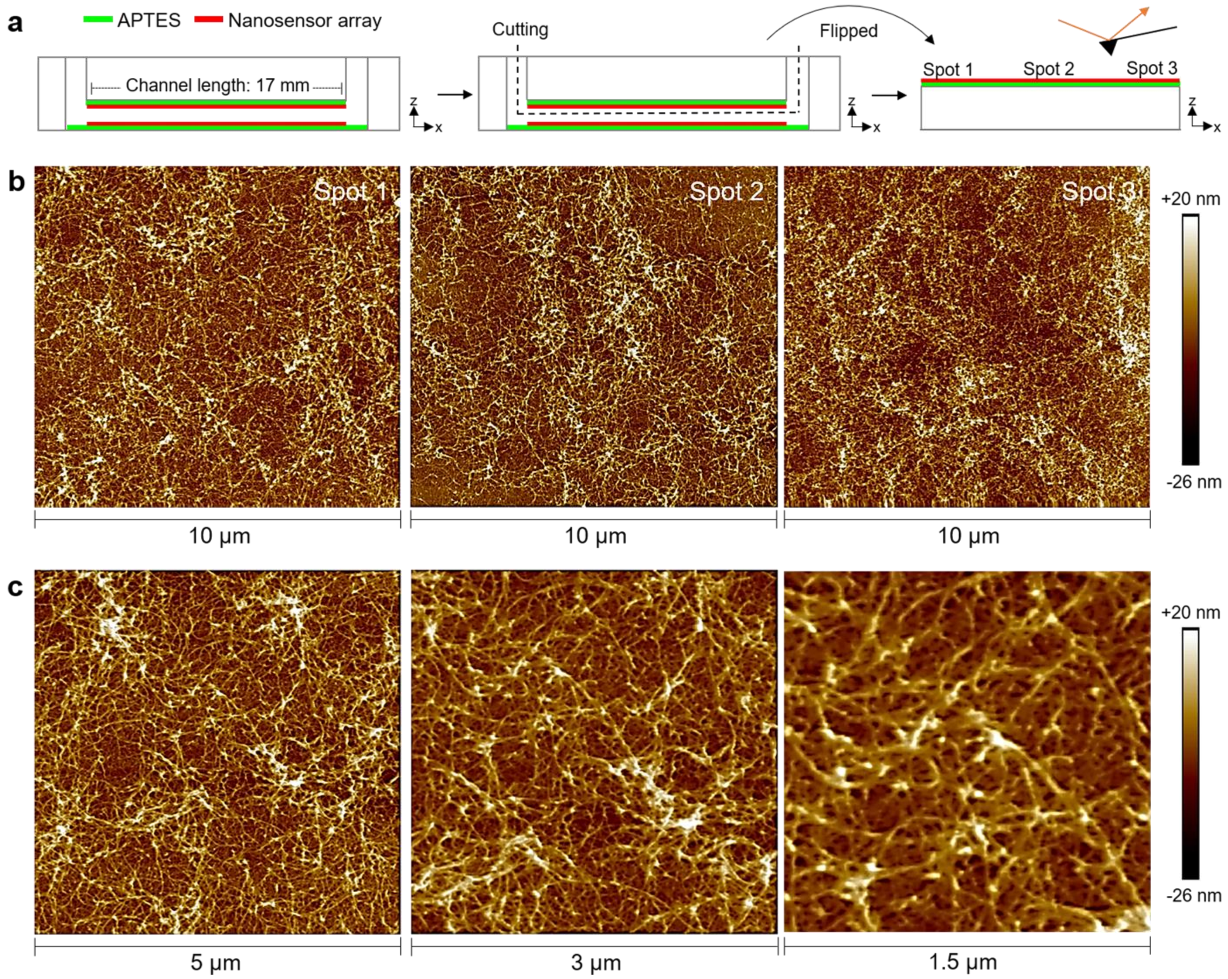

Supplementary Figure 4. AFM characterizations of nanosensor array within microfluidic channel. (a) Schematics of nanosensor array sampling of microfluidic channel for AFM measurements. (b) AFM images of spot 1 (top part), spot 2 (middle part), and spot 3 (bottom part) of single channel surface with 10 µm X 10 µm area. (c) High resolution AFM images of spot 1 with 5 µm X 5 µm, 3 µm X 3 µm area, and 1.5 µm X 1.5 µm area.

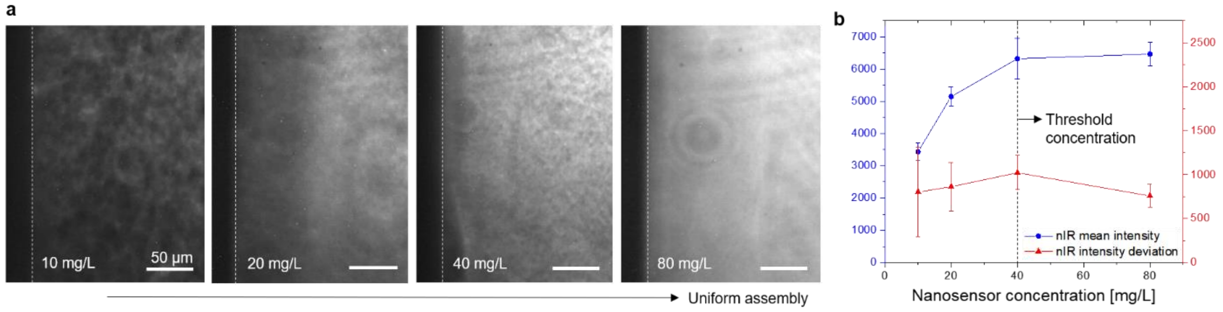

Supplementary Figure 5. Nanosensor concentration effects on ESIA process. (a) nIR images of NIM with various concentration of nanosensors (10, 20, 40, 80 mg/L). (b) nIR mean intensity and deviation profiles of NIM with various concentration of nanosensors. Data are mean (circle) ± σ (error bar) with n = 3 independent experiments.

3. Observation and characterization of nIR cellular lensing effect.

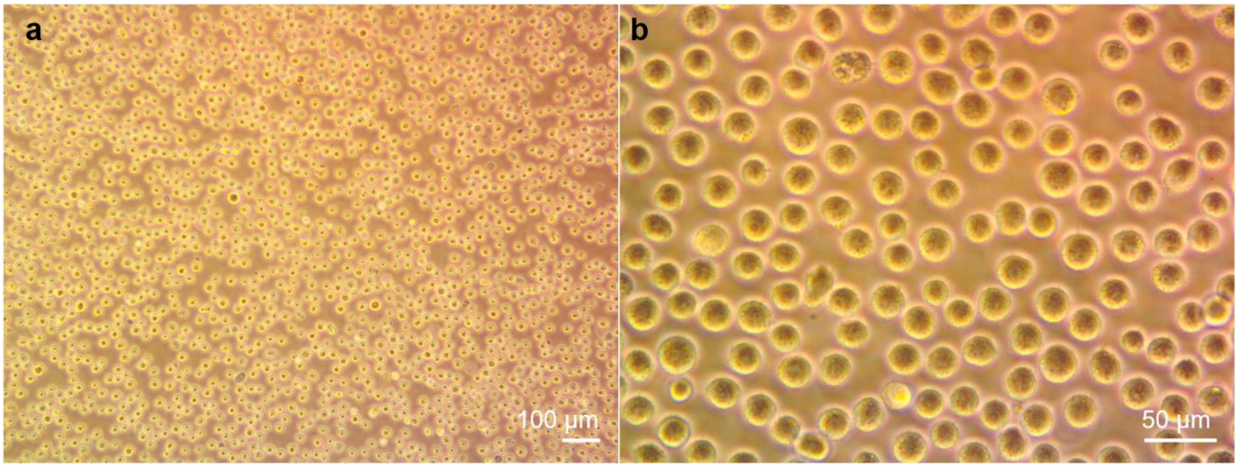

Supplementary Figure 6. (a) 10X and (b) 40X optical microscope images of human monocytes (U937) culturing for the whole experiments.

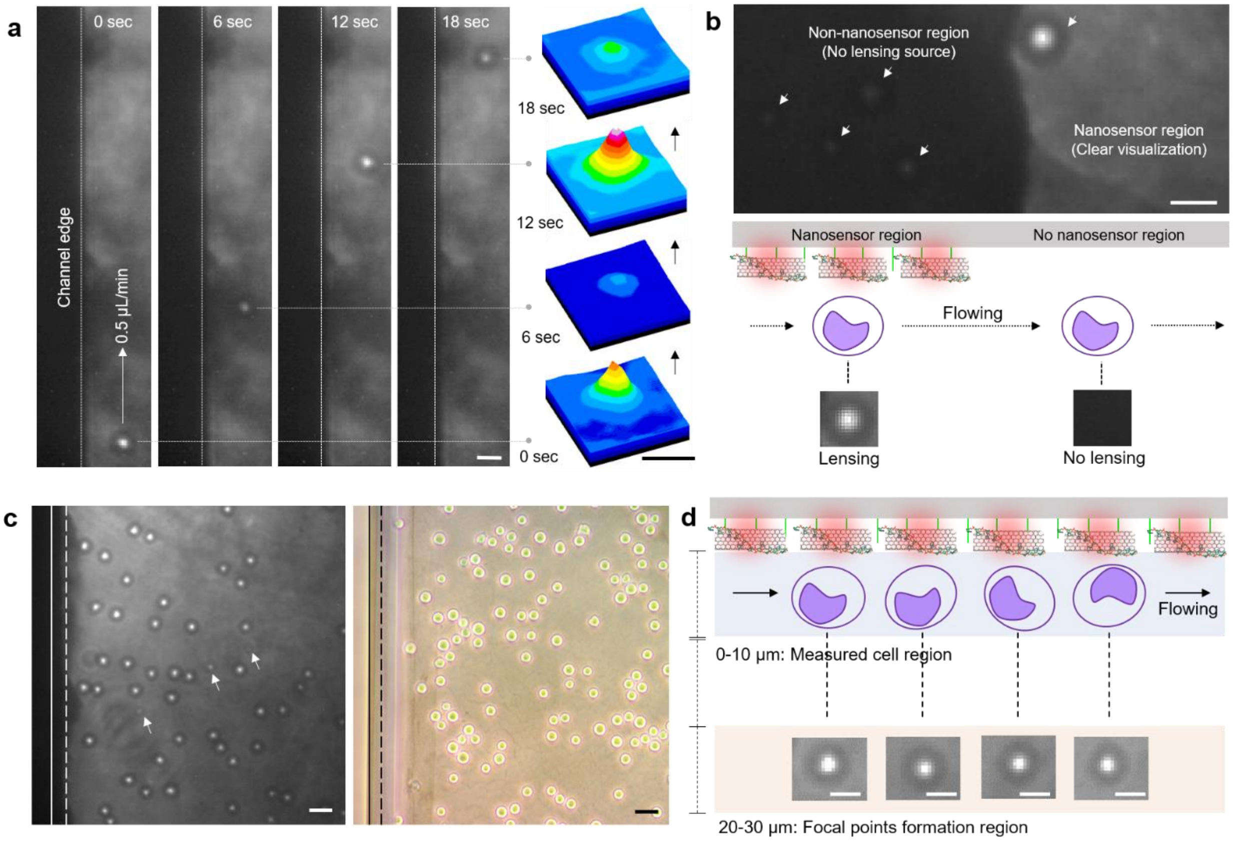

Supplementary Figure 7. Effects of underlying nanosensor array on cellular lensing. (a) Single monocyte flowing through the NIM integrated with non-uniform nanosensor array. (b) nIR image and schematics of cells flowing the nanosensor region and no nanosensor region. (c) Comparison of nIR and optical microscope image of identical NIM with same cell flowing status. Solid and dashed lines indicate the channel edge of bottom surface and top surface, respectively. White arrow indicates the cells flowing middle or bottom parts of the flow channel. (d) Schematics of cell flow and focal points formation within NIM. Scale bar: 20 μm.

To investigate the effects of underlying nanosensor array, single cell was injected through the channel having different nanosensor coating density (flow rate = 0.5 μL/min). Heterogeneous nanosensor integration was achieved by filling lower volume (*e.g.* 1 μL) of nanosensor dispersion into the pristine flow channel. nIR lensing intensity immediately changed in real-time based on different background intensity of nanosensor array even in single NIM channel (**Supplementary Figure 7a**). When the cell passes through the nanosensor array with lower nIR intensity (t = 6 and 18 sec), lensing intensity decreases and vice versa for higher nIR intensity (t = 0 and 12 sec). If the channel is not integrated with nanosensor array, cells could not be

visualized (**Supplementary Figure 7b**). Thus, we show that underlying nanosensor array of flowing cell is waveguide sources of cellular lensing effect.

Even though the identical number of cells are flowing in NIM, only ~50% of the total cells were visualized with lensing effect compared to optical microscope measurement (**Supplementary Figure 7c**). In our experiments, cell diameter is around 10 - 20 μm, channel height is 100 μm, and flowing is based on laminar flow ($Re \sim 10^{-3}$). Accordingly, cell populations should form a few layers of flowing between top and bottom surfaces of NIM. If we use optical microscope to observe the cells, which is based on the visible light scattering from bottom surface, all flowing cells in NIM could be observed. However, for the nIR measurements, fluorescence from top nanosensor array is light source for cell visualizations with cellular lensing effect. Thus, only the cells flowing near the top surfaces (0 - 10 μm) could be visualized with underlying nanosensor array having 20 - 30 μm focal distance as demonstrated in FDTD numerical calculations (**Supplementary Figure 7d**). Cells flowing middle or bottom of the channel were not visualized as the increased distance from the nanosensor nIR source decreases light collection into the cell microlens (as indicated with white arrow of **Supplementary Figure 7c**).

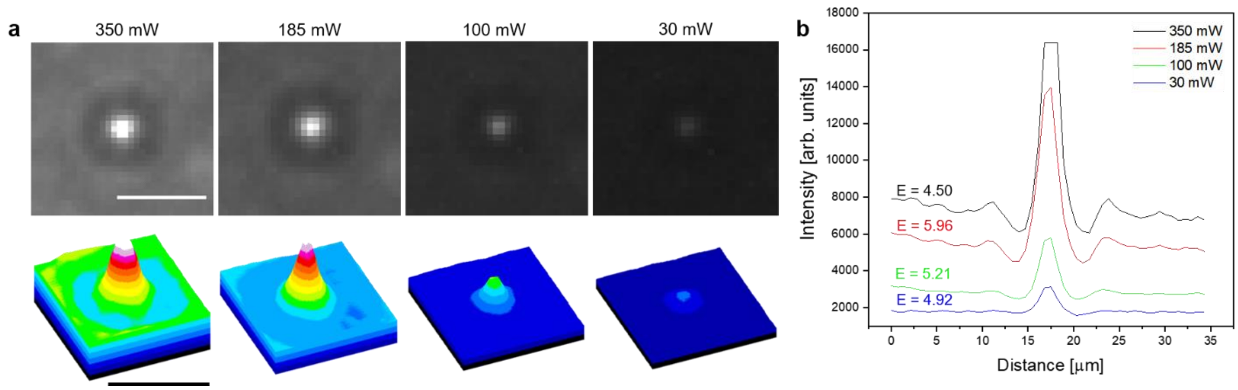

Supplementary Figure 8. Effects of excitation laser power control on cellular lensing. (a) nIR images with 3D intensity mapping and (b) cross-sectional intensity profiles of identical single cell with various power (30 to 350 mW) of excitation light (531 nm). Cellular lensing effect were observed for all range of excitation powers and lens intensity (I_0) was drastically enhanced with increased laser power. There was optimum laser power (185 mW), in which enhancement factor of the cell was highest (5.96). Accordingly, excitation power of 185 mW was applied for all following experiments.

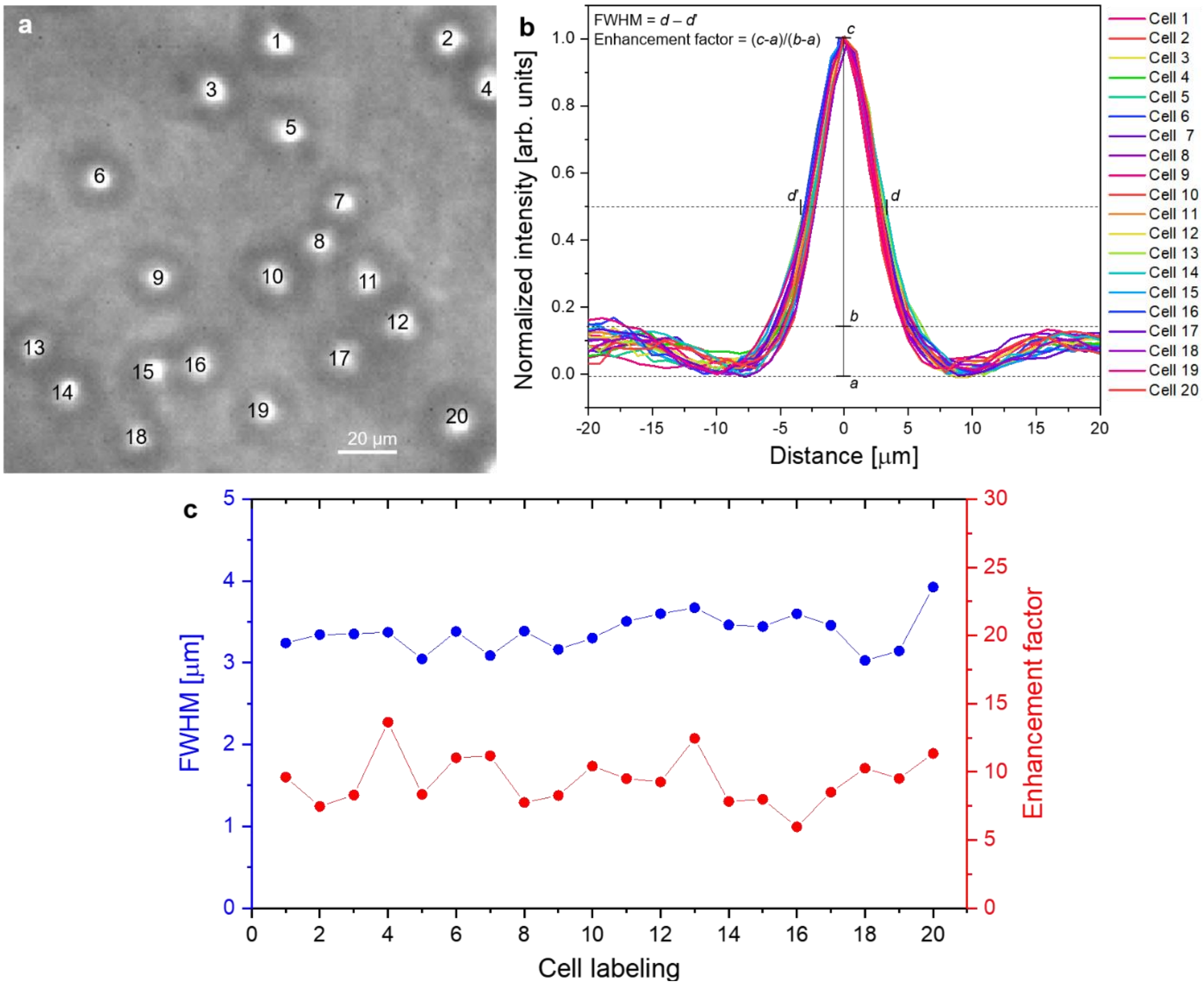

Supplementary Figure 9. nIR lensing profiles of multiple cells with identical cell status (human monocyte (U937), non-activated, $n_{\mathrm{cell}} = 20$ in single experiment). (a) nIR images of the cells in NIM and (b) their lensing profiles. (c) Calculated full-width half-maximum (FWHM) and enhancement factors of the cells. Cells showed average FWHM (3.37 ± 0.22 μm) and enhancement factor (9.43 ± 1.86) with low deviations (<10 %) indicating that this lensing effect is reliable and specific to certain cell properties.

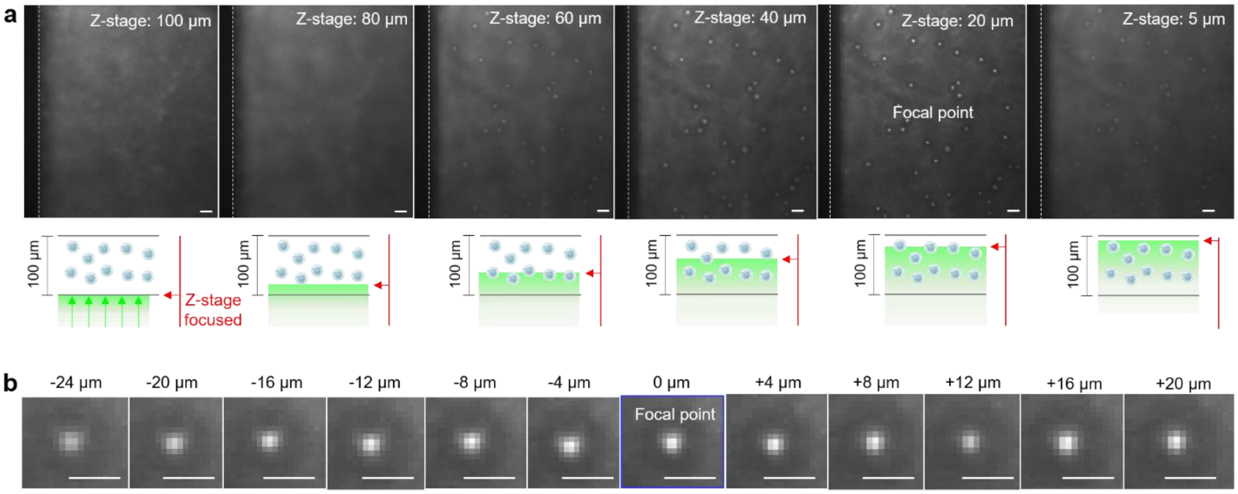

Supplementary Figure 10. Z-stage control experiments for investigating focal point formation of nIR lensing effect. (a) nIR images of stationary cells with Z-stage height of 100, 80, 60, 40, 20, and 5 µm from top surface of NIM. (b) Magnified nIR image of stationary single cell with focusing point resolution of 4 µm. Scale bar: 20 µm.

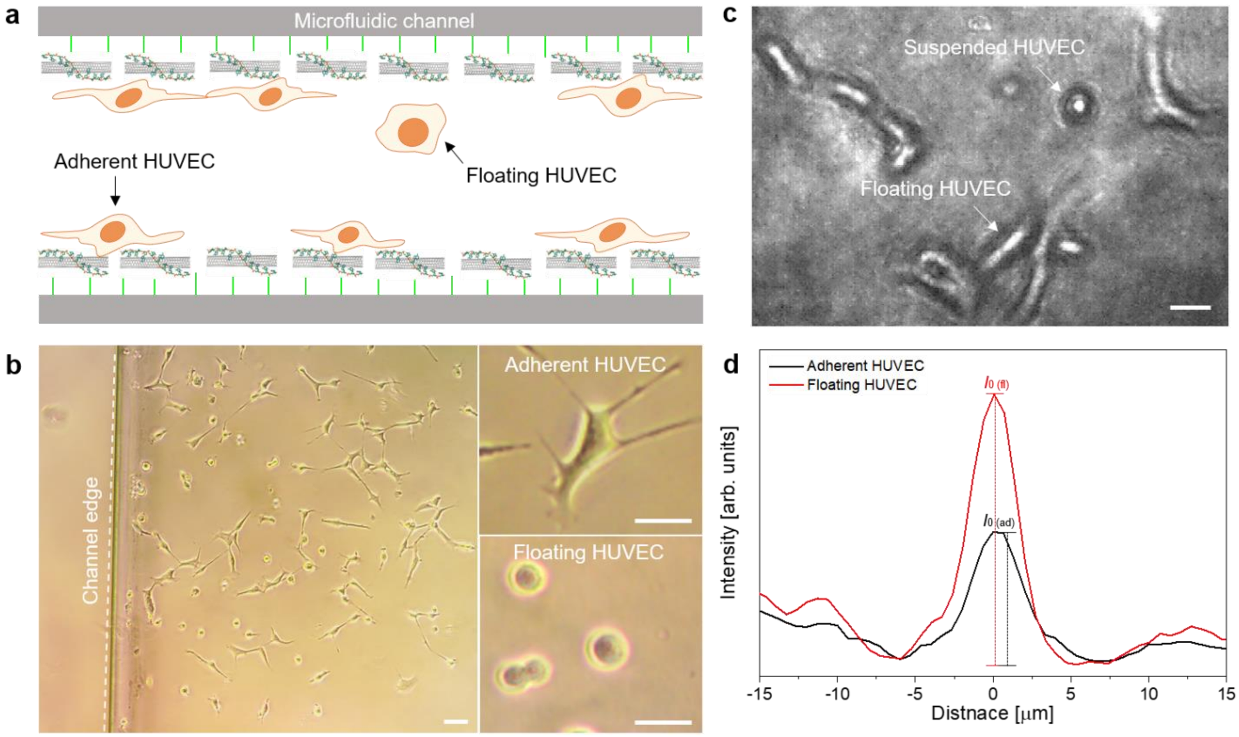

Supplementary Figure 11. nIR lensing effect of adherent cells on nanosensor array. (a) Schematics of human umbilical vein cells (HUVECs) culturing in NIM. (b) Optical microscope and (c) nIR images HUVECs cultured on NIM with cellular lensing effect. (d) Comparison of nIR lensing profiles from adherent and floating HUVECs (cells were indicated in (c) with white arrow). Scale bar: 20 μm.

In order to investigate the nIR lensing effects of adherent cell type, HUVECs were cultured in NIM (**Supplementary Figure 11a**). Optical microscope images show that HUVECs were well grown on the nanosensor array of the NIM (**Supplementary Figure 11b**). Not only for adhered cell status, suspended HUVECs were also observed. When the channel was measured by nIR instrumentation, adherent HUVECs in the channel were also clearly visualized with nIR lensing effect along the elongated cell shape (**Supplementary Figure 11c**). nIR lensing profiles of adherent HUVECs were smaller than those of suspended flowing HUVECs since the depth of the lens is smaller (**Supplementary Figure 11d**). Thus, we show that nIR lensing effects can be universally applied to both adherent and suspended cells.

4. FDTD numerical modeling of photonic nanojet effect of cells.

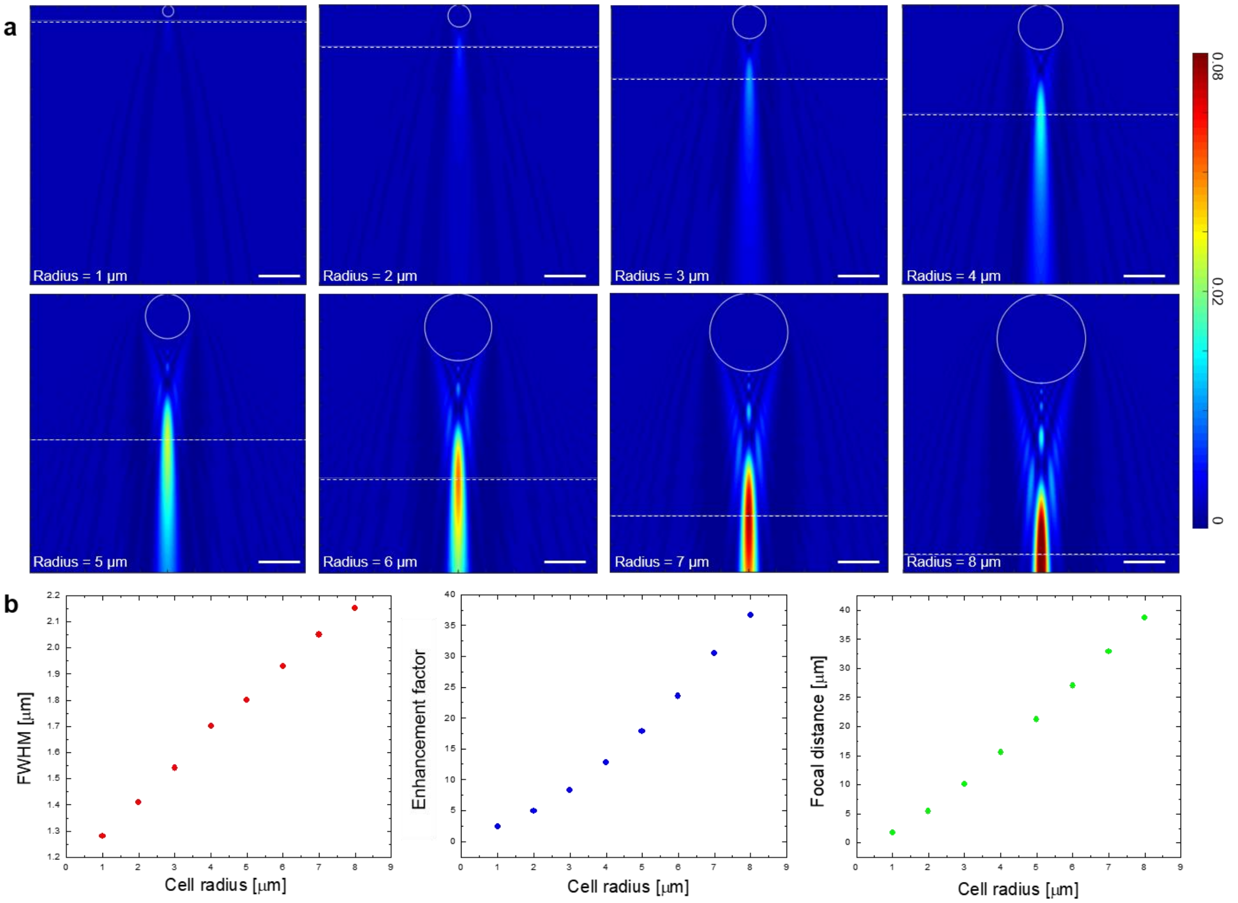

Supplementary Figure 12. FDTD modeling results for nIR photonic nanojet effects with various cell size (radius: 1, 2, 3, 4, 5, 6, 7, and 8 µm with n_c/n_m = 1.05 and eccentricity = 1). (a) Wavelength power profiles of the cell with various cell size. White dashed lines and solid circles indicate the focal points and cell model, respectively. Scale bar: 10 µm. (b) FWHM, enhancement factor, and focal distance of the cell with various cell size.

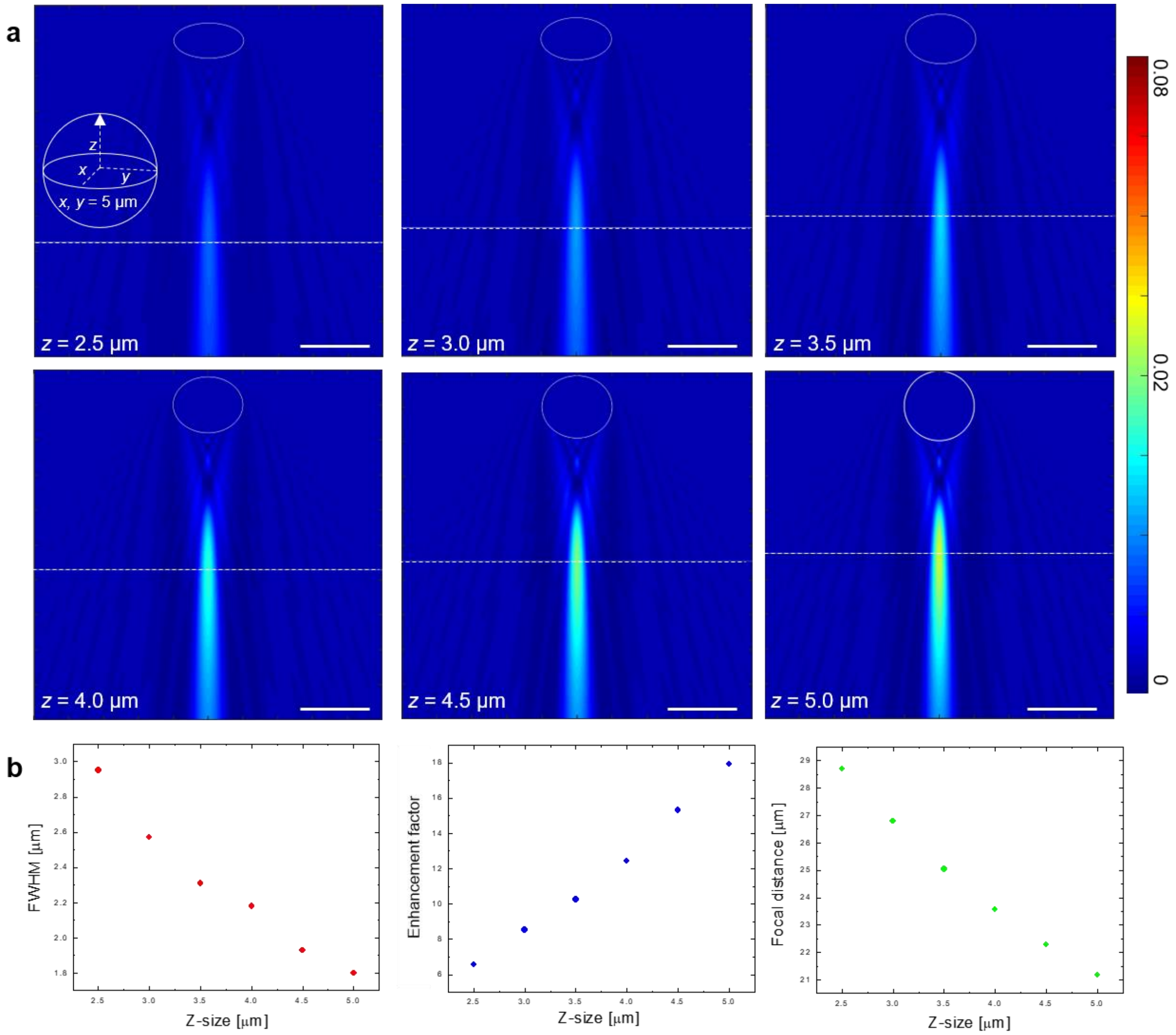

Supplementary Figure 13. FDTD modeling results for nIR photonic nanojet effects with various cell eccentricity (z-axis distance: 2.5, 3, 3.5, 4, 4.5, and 5.0 µm with cell radius 5 µm and n_c/n_m = 1.05). (a) Wavelength power profiles of the cell with various cell eccentricity. White dashed lines and solid circles indicate the focal points and cell model, respectively. Scale bar: 10 µm. (b) FWHM, enhancement factor, and focal distance of the cell with various cell eccentricity.

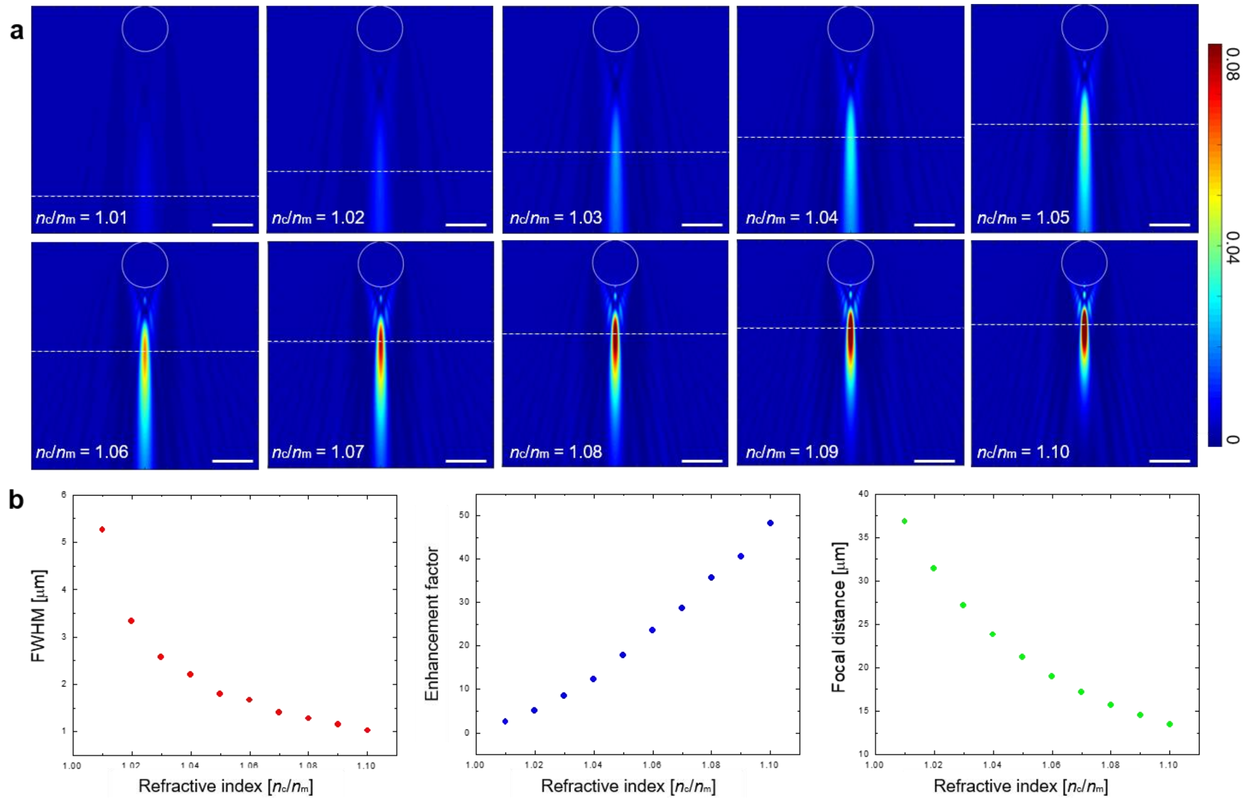

Supplementary Figure 14. FDTD modeling results for nIR photonic nanojet effects with various cell RI (n_c/n_m: 1.01, 1.02, 1.03, 1.04, 1.05, 1.06, 1.07, 1.08, 1.09, and 1.10 with cell radius 5 µm and eccentricity = 1). (a) Wavelength power profiles of the cell with various cell RI. White dashed lines and solid circles indicate the focal points and cell model, respectively. Scale bar: 10 µm. (b) FWHM, enhancement factor, and focal distance of the cell with various cell RI.

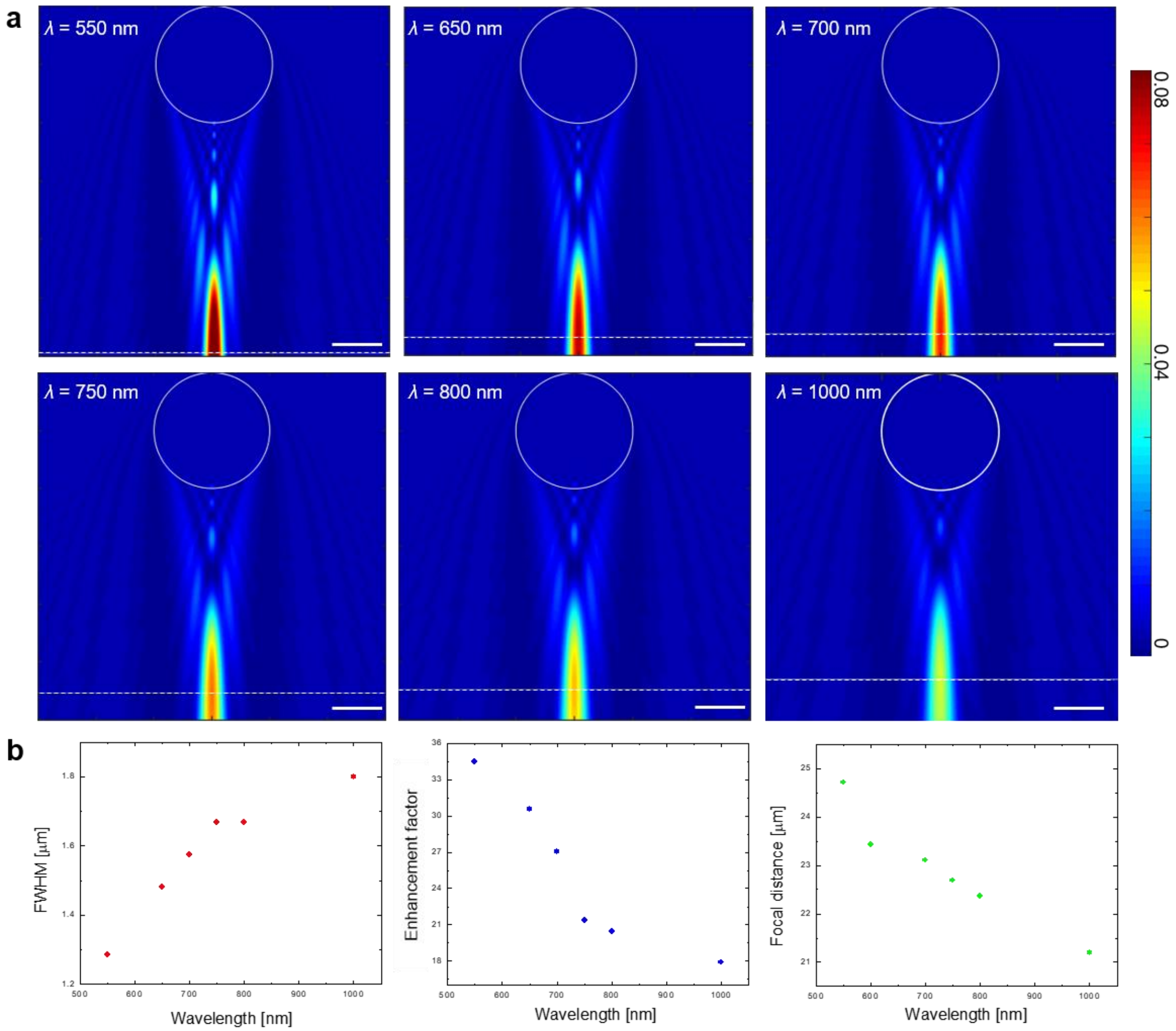

Supplementary Figure 15. FDTD modeling results for nIR photonic nanojet effects with various wavelength of light source (λ: 550, 650, 700, 750, 800, and 1000 nm with cell radius 5 μm and eccentricity = 1). (a) Wavelength power profiles of the cell with various beam wavelength. White dashed lines and solid circles indicate the focal points and cell model, respectively. Scale bar: 5 μm. (b) FWHM, enhancement factor, and focal distance of the cell with various beam wavelength.

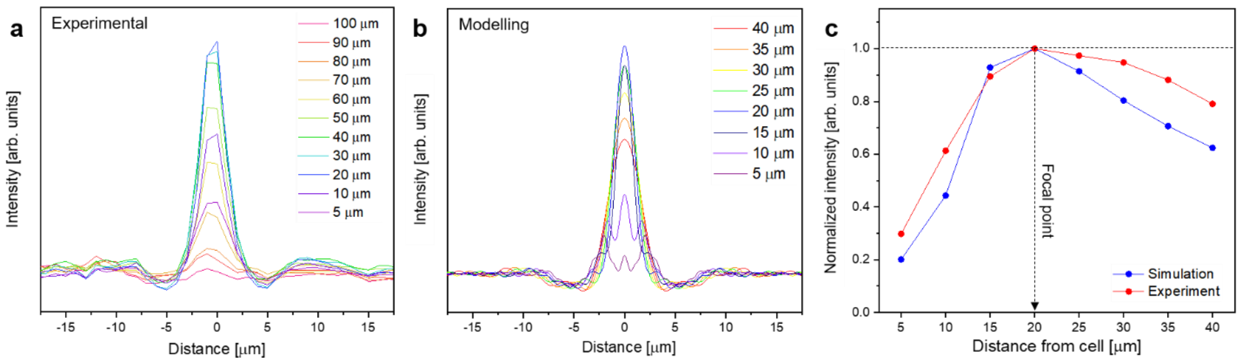

Supplementary Figure 16. nIR lensing profiles of the cell with different focusing points. Z-stage controller was integrated with objective of our hyperspectral instrument to precisely control the focusing depth. (a) Experimental and (b) FDTD simulation data for nIR lensing profiles with different focusing points (5 to 100 μm). (c) Normalized lensing intensities (I_0) plot of the cell with various focusing points (5 to 40 μm). Both experimental and simulations profiles showed 20 μm as focal point indicating that cellular lensing images were formed at 20 μm distant points from cells, which were flowing just below (0 - 10 μm) the top surface of NIM.

5. Chemical efflux monitoring using nIR lensing effect.

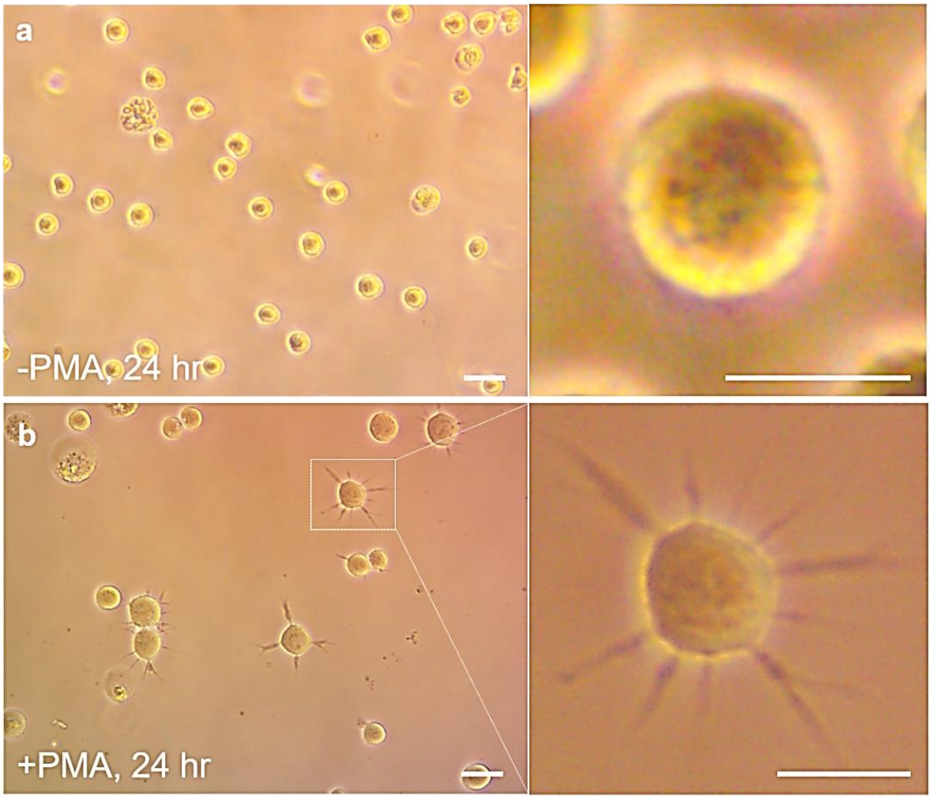

Supplementary Figure 17. Immune activation of human monocyte (U937) with phorbol 12-myristate 13-acetat (PMA) treatments. Optical microscope images of human monocytes (a) without (-PMA, 24 hr) and (b) with (+PMA, 24 hr) PMA treatment (5 μg/mL). Monocytes showed prominent morphological transformation from suspended circular shape into macrophage-like attached elongation. Scale bar: 20 μm.

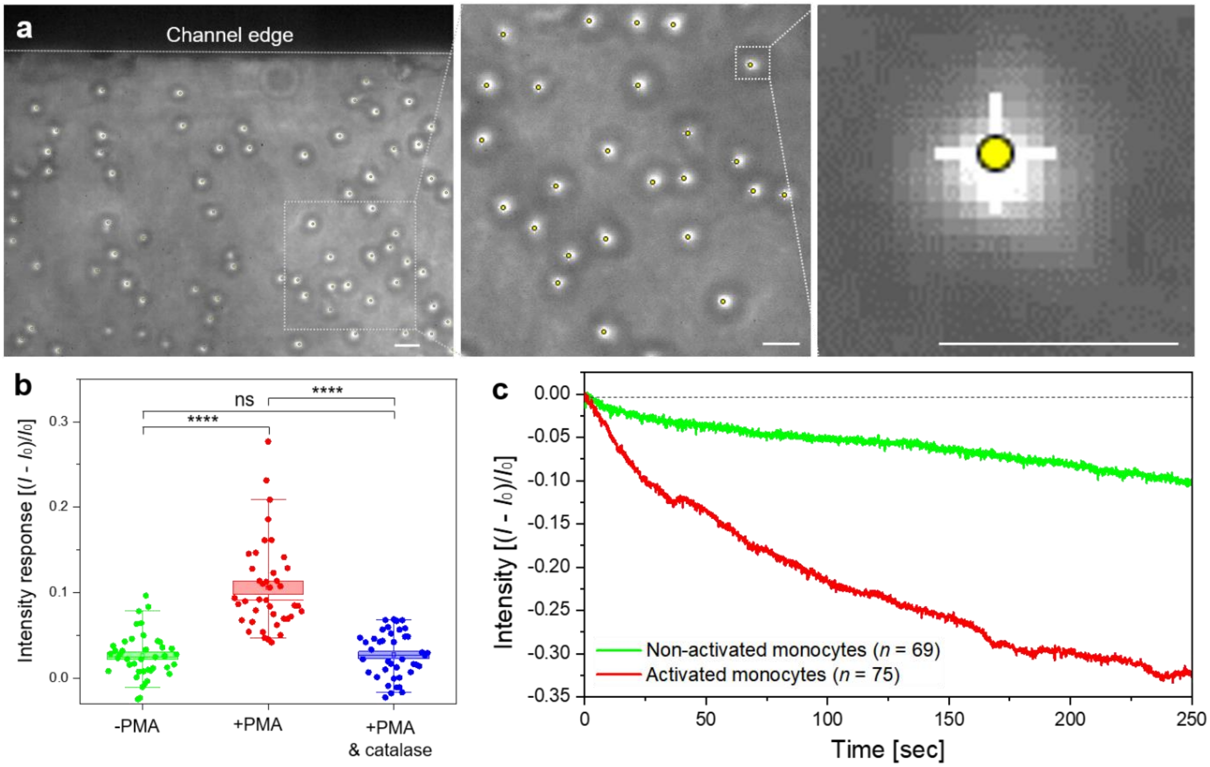

Supplementary Figure 18. Real-time monitoring of nIR signal from multiple monocytes. (a) nIR images of measured cell group, which is automatically designated by lensing intensities (I_0). With cellular lensing effect, all the cells were clearly identified automatically. (b) Sensor response statistics of the cells with different activation states. Data are mean ± standard error (bounds of box) and 5 - 95 % range (whisker), with n_{cell} = 41 from n = 3 biologically independent samples. p values were calculated using one-way analysis of variance (ANOVA), followed by Tukey analysis (****$p < 0.0001$, ns = 0.9934). (c) Averaged nIR sensor response from total cells in each snap shot (n = 69 for -PMA and n = 75 for +PMA). +PMA cells showed much higher sensor response than -PMA cells. Scale bar: 20 μm.

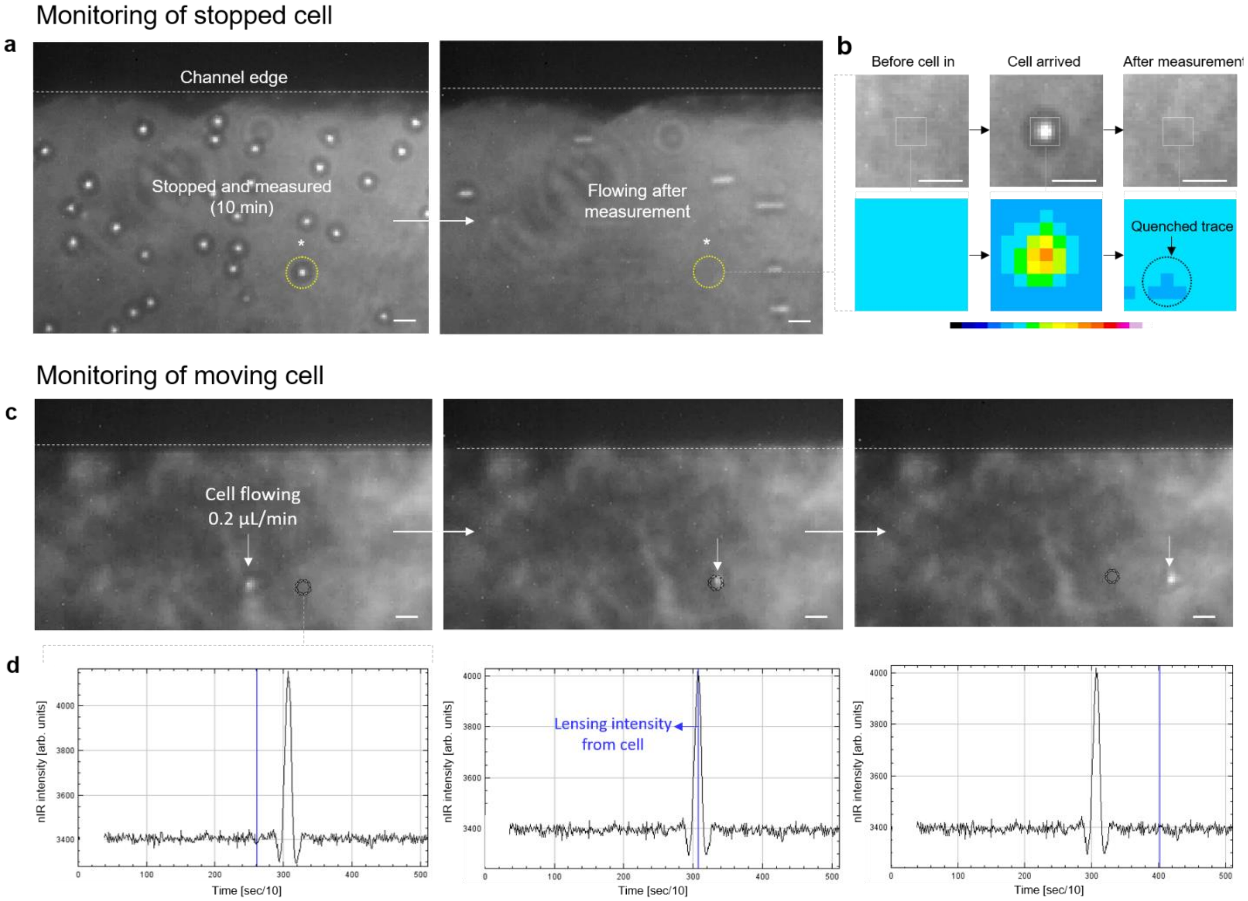

Supplementary Figure 19. Monitoring of H_2O_2 efflux traces from the stopped and moving cells. (a) nIR images of stopped and measured cells for 10 min. (b) Quenched trace of nanosensor array by H_2O_2 efflux from measured cell. (c) nIR images of moving cell with 0.2 μL/min flow. (d) Time-series cross-sectional nIR intensity profile of the fixed measurement spot among cell flowing trace. There are no quenched spot or signal variations during cell flow since H_2O_2 efflux from cell was hard to be interacting with underlying nanosensor array. Scale bar: 20 μm.

Supplementary Note 1. H_2O_2 efflux wave modeling.

We used 3D diffusion equation with sources and sinks to model real-time H_2O_2 efflux. Here, H_2O_2 diffusion is assumed to be isotropic, instantaneous (at $t = 0$), localized ((x, y, z) = (0, 0, 0) = cell core) and temporal decaying, and the concentration C does not depend on the direction with respect to the coordinate axes. The chemical flux quantity has three components (east-west, north-south, and up-down) in a vector form as

$$\vec{q} = (q_x, q_y, q_z) \quad \text{(Supplementary Equation 1)}$$

$$q_x = -D\frac{\partial C}{\partial x}, q_y = -D\frac{\partial C}{\partial y}, q_z = -D\frac{\partial C}{\partial z} \quad \text{(Supplementary Equation 2)}$$

where C is H_2O_2 concentration at (x, y, z, t) and D is diffusion coefficient of H_2O_2 ($1.5 \cdot 10^{-5}$ $cm^2 \cdot sec^{-1}$). Putting it all together and using vector notation, we can write:

$$\vec{q} = -D\left(\frac{\partial c}{\partial x}, \frac{\partial c}{\partial y}, \frac{\partial c}{\partial z}\right) = -D\,\vec{\nabla}C \quad \text{(Supplementary Equation 3)}$$

where $\vec{\nabla}$ is the gradient operator. All mass of the chemical substances mush be accounted for

$$\text{Rate of accumulation} = \sum \text{import} + \sum \text{export} \quad \text{(Supplementary Equation 4)}$$

For an infinitesimal 3D box of volume dxdydz, Supplementary Equation 4 becomes

$$\mathrm{d}x\mathrm{d}y\mathrm{d}z\,\frac{\partial C}{\partial t} = -\left(\frac{\partial q_x}{\partial x} + \frac{\partial q_y}{\partial x} + \frac{\partial q_z}{\partial x}\right)\mathrm{d}x\mathrm{d}y\mathrm{d}z - KC \quad \text{(Supplementary Equation 5)}$$

where K is decay constant of H_2O_2. Here, decay constant can be related to the cellular half-life of H_2O_2 ($t_{1/2} = 10^{-3}$ sec) for first order reaction in the following way

$$\ln\frac{[H_2O_2]_t}{[H_2O_2]_0} = \ln\frac{\frac{1}{2}[H_2O_2]_0}{[H_2O_2]_0} \quad \text{(Supplementary Equation 6)}$$

$$\ln\frac{1}{2} = -Kt_{1/2} \quad \text{(Supplementary Equation 7)}$$

Therefore, $K = 6.93 \cdot 10^{-4}$ sec^{-1}. With Supplementary Equation 3, Supplementary Equation 5 reduces to

$$\frac{\partial C}{\partial t} = D\nabla^2 C - KC \quad \text{(Supplementary Equation 8)}$$

based on Fick's second law where ∇^2 is the Laplace operator. Initial and boundary conditions are as below

$$C = C_0(x, y, z) = M\delta(x, y, z) \text{ at } t = 0 \quad \text{(Supplementary Equation 9)}$$

$$\lim_{x,y,z\to\infty} C = \lim_{x,y,z\to-\infty} C = 0 \quad \text{(Supplementary Equation 10)}$$

where *M* is the total units of the chemical substances (*e.g.* mole), and δ is the Dirac function (δ = 0 for *x*, *y*, *z* $\neq$ 0, δ = +∞ at *x*, *y*, *z* = 0, and area under the infinitely tall and infinitely narrow peaks are unity). Finally, with the size/shape factors structure and initial/boundary conditions, Supplementary Equation 8 can be solved into H_2O_2 efflux diffusion model as below

$$C(x, y, z, t) = \frac{M}{\left(\sqrt{4\pi Dt}\right)^3} \exp\left(-\frac{x^2+y^2+z^2}{4Dt} - Kt\right) \quad \text{(Supplementary Equation 11)}$$

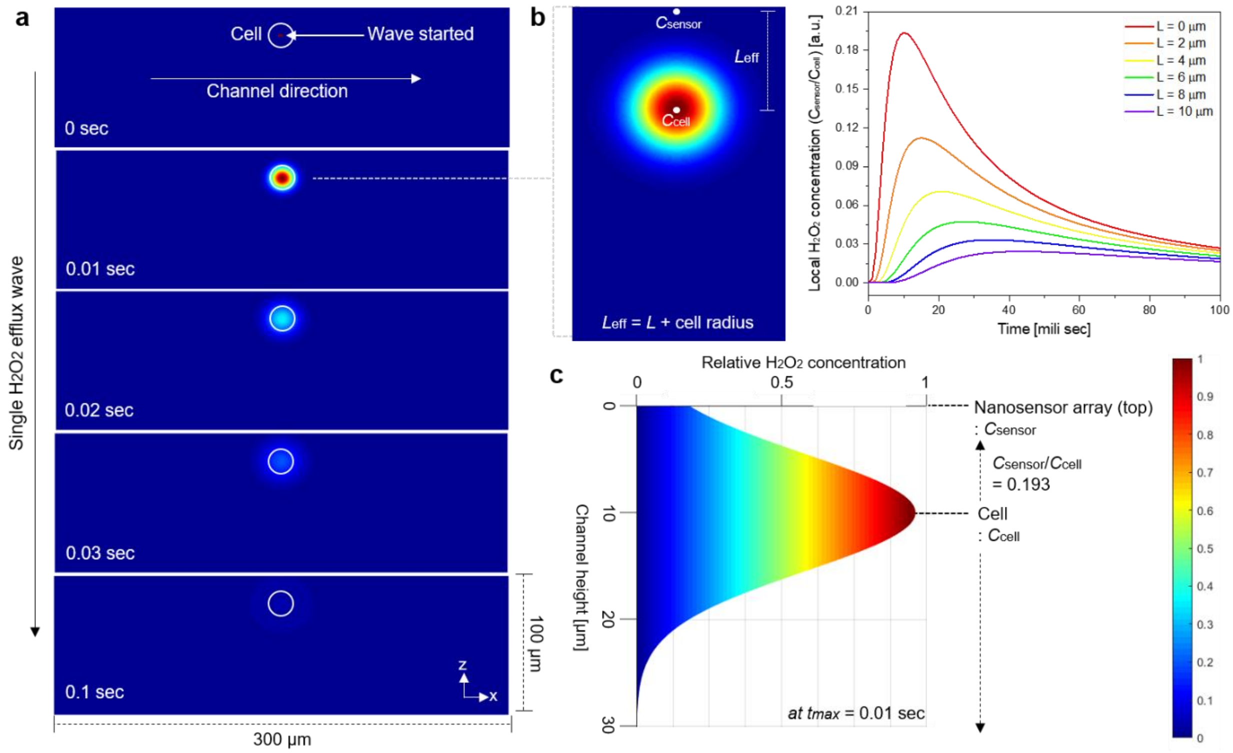

Supplementary Figure 20. Numerical modeling of H_2O_2 efflux wave from single cell in NIM. (a) Time-series (0, 0.01, 0.02, 0.03, 0.1 sec) diffusion behavior of instantaneous H_2O_2 efflux wave in flow channel. Cell is located in 10 µm below the nanosensor array. Profiles were from *x*-*z* plane and *y* = 0. (b) Calculated dynamics of H_2O_2 concentration at nanosensor array (C_{sensor}/C_{cell}) with various location from cell (L = 0, 2, 4, 6, 8, and 10 µm). (c) Gradient profile (along Z-axis) of H_2O_2 local concentration between cell and nanosensor array (L = 0 µm, t_{max} = 0.01 sec). Color bar indicates relative H_2O_2 concentration normalized by maximum H_2O_2 concentration at source (C_{cell} at t_{max}).

Using Supplementary Equation 11 with D_{H2O2} and K_{H2O2}, 3D diffusion profiles of chemical efflux wave from single cell were calculated in time-series. Here, *x*-*z* plane profiles at *y* = 0 were calculated to highlight cross-sectional diffusion wave of stationary cell in flow channel. Single H_2O_2 efflux wave was plotted with 3D surf function of MATLAB (Natick, MA) (**Supplementary Figure 20a**). Assuming H_2O_2 efflux started within the cell, efflux cloud was fastly diffused throughout the near cell region (around 35 µm region) within 0.01-0.02 sec and diminished gradually until 0.1 sec. Based on the model, real-time H_2O_2 concentration variation at nearest nanosensor array ($C_{sensor} = C\ (0, 0, L_{eff})$) was calculated (**Supplementary Figure 20b**).

Here, L_{eff} is effective distance between source and nanosensor array (L_{eff} = L + cell radius), where L was assumed to be below 10 μm, as demonstrated in **Figure 3e**. For higher L_{eff}, it takes longer time to reach the nanosensor array (0.01 → 0.04 sec) and maximum C_{cell} is lower. For L_{eff} = 10 μm, H_2O_2 efflux wave reached the nearest nanosensor array in shortest time (t_{max} = 0.01 sec) with highest concentration (C_{sensor}) and gradient between C_{sensor} and C_{cell} was 0.193 (**Supplementary Figure 20c**). We assumed that our real-time measurements of cellular lensing intensities were from this highest H_2O_2 efflux wave reached on nanosensor array (L_{eff} = 10 μm, t_{max} = 0.01 sec). Accordingly, H_2O_2 concentrations calculated from nIR signals of nanosensor array were about 19.3% diffused amounts of original cell concentration.

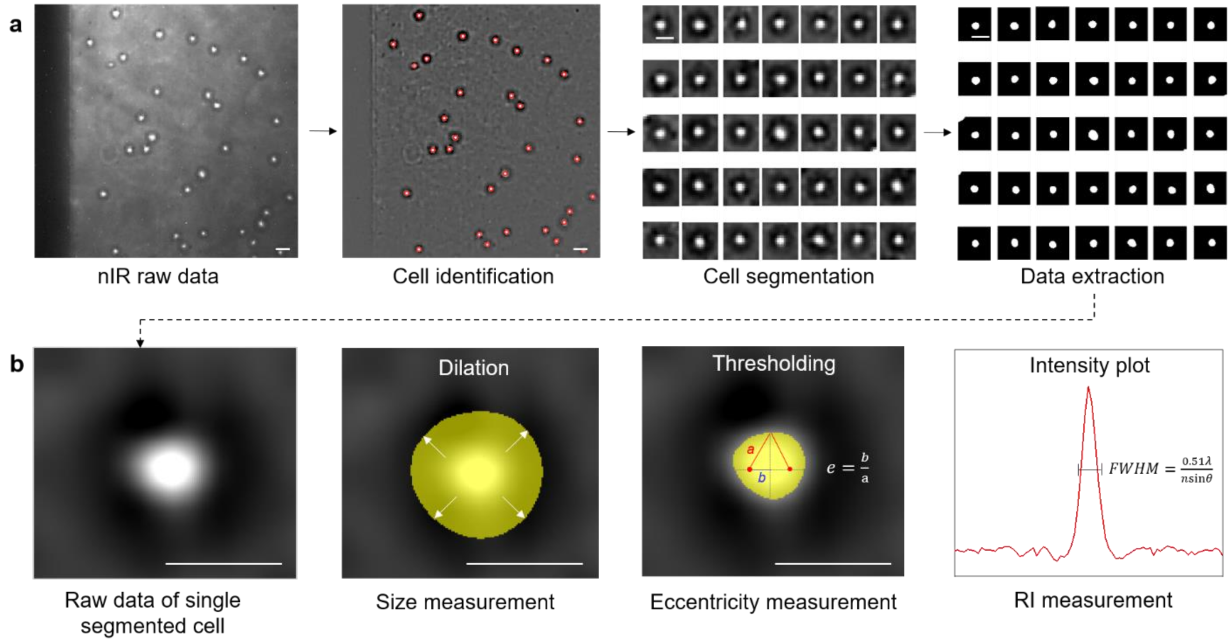

Supplementary Figure 21. Automated image analysis program using MATLAB for NCC data extraction. (a) Each single cell is identified and segmented from nIR raw data. (b) Cell size, eccentricity and RI of segmented single cell were calculated with dilation, thresholding, and FWHM calculations. Scale bar: 20 µm.

Automatic nIR image analysis and quantitation was performed in MATLAB with the steps detailed below. Cell identification is performed by taking 1 frame of the nIR video (5000 sec, 0.1 sec of exposure time) convolving with a Laplacian of a Gaussian filter, and then thresholded by the user for each experiment batch (**Supplementary Figure 21a**). For each cell, the nIR image is then interpolated. Using the peak and trough of the nIR lensing spot, the image is normalized, and then statistics such as area and eccentricity are evaluated with dilation and thresholding of "regionprops" function (**Supplementary Figure 21b**). The area values are dilated appropriately to coincide with the photonic nanojet model. RI of cell is calculated from FWHM-numerical equation for spherical lens point spread functions (PSF). To avoid excess data interpolation, camera pixel intensities are used for subsequent analysis. The cell lensing intensity is found by choosing the camera pixel closest to the centroid. To calculate background, 16 pixels

outside of the secondary peak of the lensing effect is chosen. Outliers are then removed, and background traces are averaged to use as normalization for the centroid intensity traces.

6. 3D cytometry plots.

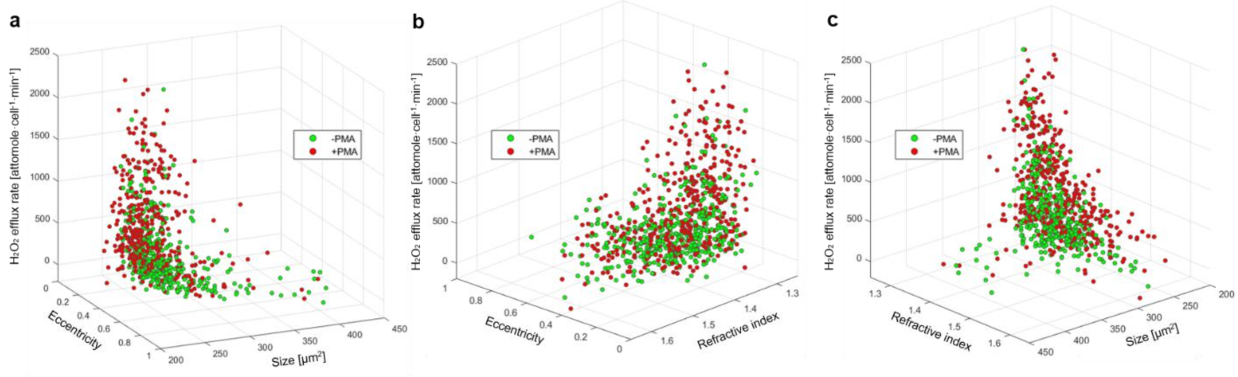

Supplementary Figure 22. 3D cytometry plots of -PMA and +PMA human monocyte populations. (a) H_2O_2 efflux rate *vs* eccentricity *vs* size. (b) H_2O_2 efflux rate *vs* eccentricity *vs* RI. (c) H_2O_2 efflux rate *vs* RI *vs* size. Data are n_{cell} = 413 for -PMA, n_{cell} = 414 for +PMA from n = 6 biologically independent samples.

7. 2D Kernel density estimations.

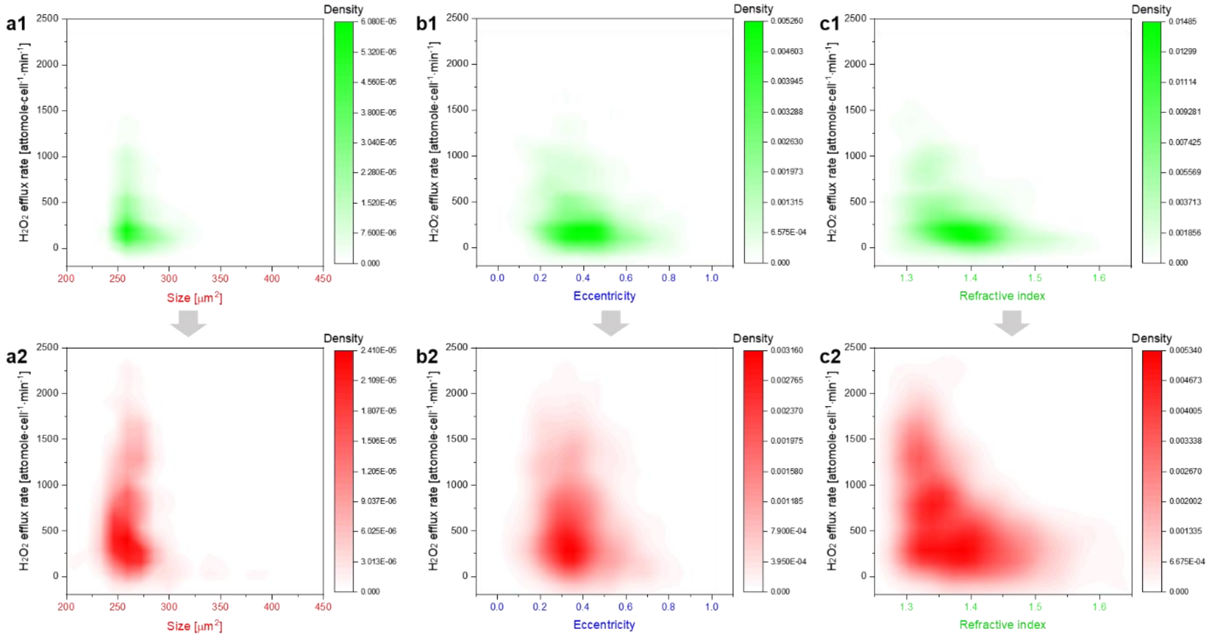

Supplementary Figure 23. 2D Kernel density estimations of cytometry plots in **Figure 5c-e**. Non-activated monocytes (-PMA) density profiles for (a1) H_2O_2 efflux rate *vs* size, (b) H_2O_2 efflux rate *vs* eccentricity, and (c1) H_2O_2 efflux rate *vs* RI. Activated monocytes (+PMA) density profiles for (a2) H_2O_2 efflux rate *vs* size, (b2) H_2O_2 efflux rate *vs* eccentricity, and (c2) H_2O_2 efflux rate *vs* RI. Bivariate Kernel density estimator was used and number of grid points in X/Y is 32. Kernel density estimations clearly show that non-activated and activated monocytes distinct H_2O_2 efflux behavior in terms of various biophysical parameters.

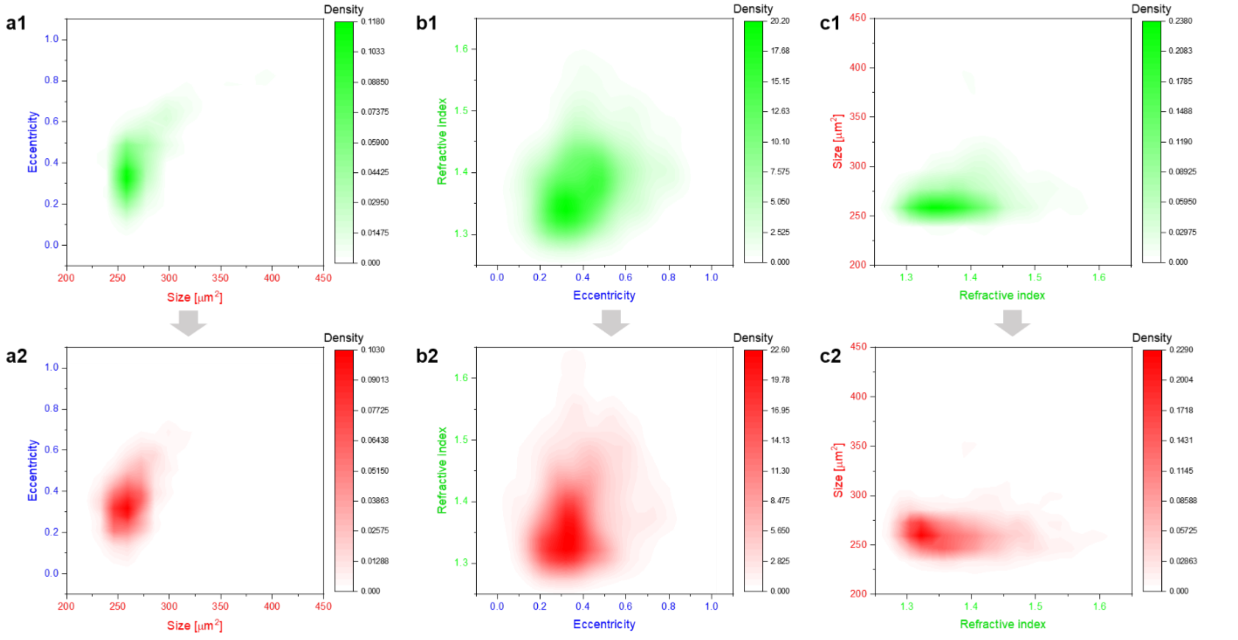

Supplementary Figure 24. 2D Kernel density estimations of cytometry plots in **Figure 5g-i**. Non-activated monocytes (-PMA) density profiles for (a1) eccentricity *vs* size, (b) RI *vs* eccentricity, and (c1) size *vs* RI. Activated monocytes (+PMA) density profiles for (a2) eccentricity *vs* size, (b2) RI *vs* eccentricity, and (c2) size *vs* RI. Bivariate Kernel density estimator was used and number of grid points in X/Y is 32.

8. NCC data comparison with commercial H_2O_2 assay kit.

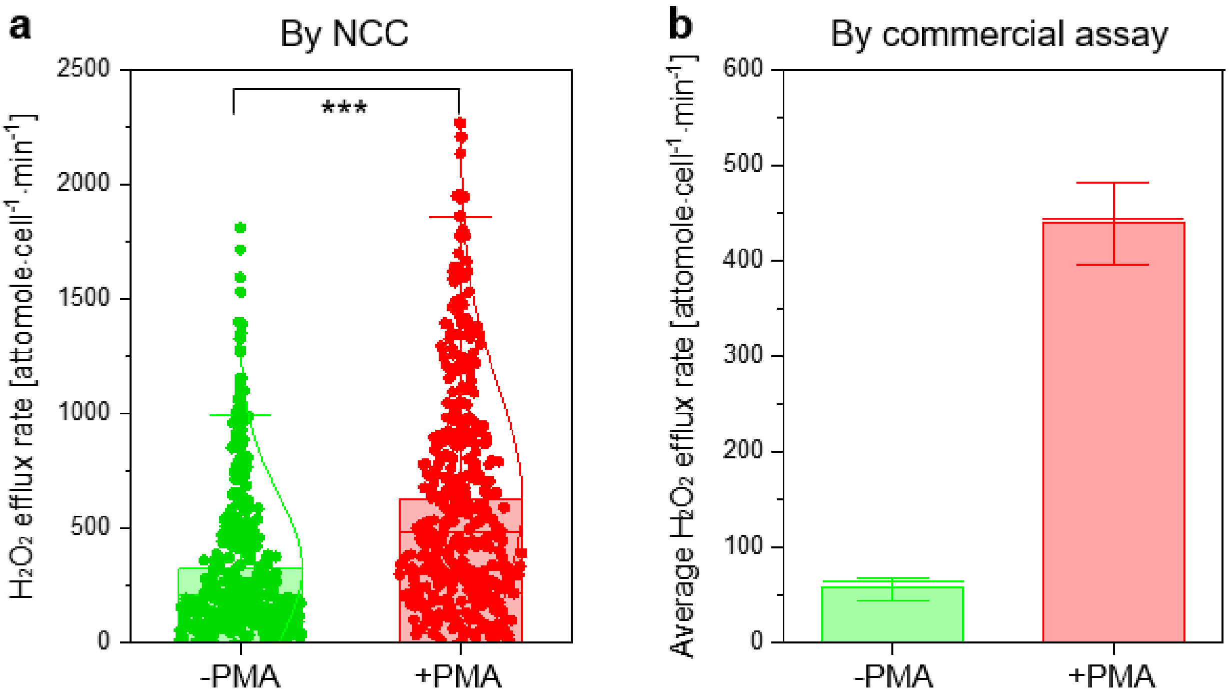

Supplementary Figure 25. Comparison of NCC and commercial assay. Statistics from measurement by (a) NCC and (b) commercial assay kit (Amplex UtraRed assay). Data are mean (box) ± σ (error bar), with n = 3 biologically independent samples. p values were calculated using one-way ANOVA, followed by Tukey analysis (***p (= 0.00329) < 0.005).

Commercial H_2O_2 assay was measured via horseradish peroxidase based Amplex UltraRed reagent protocol (Thermofisher, A36006). 50 μL of cells (-PMA and +PMA) were mixed with the 50 μL Amplex UltraRed reagent working solution at 1:1 ratio and incubated 15-30 min. Then, fluorescence was measured after 140 min with excitation and emission channels set at 490 and 585 nm. A standard curve for H_2O_2 was developed along with the samples to ensure the signals from the samples were within the linear range and converted for quantification of H_2O_2.

9. Throughput calculation.

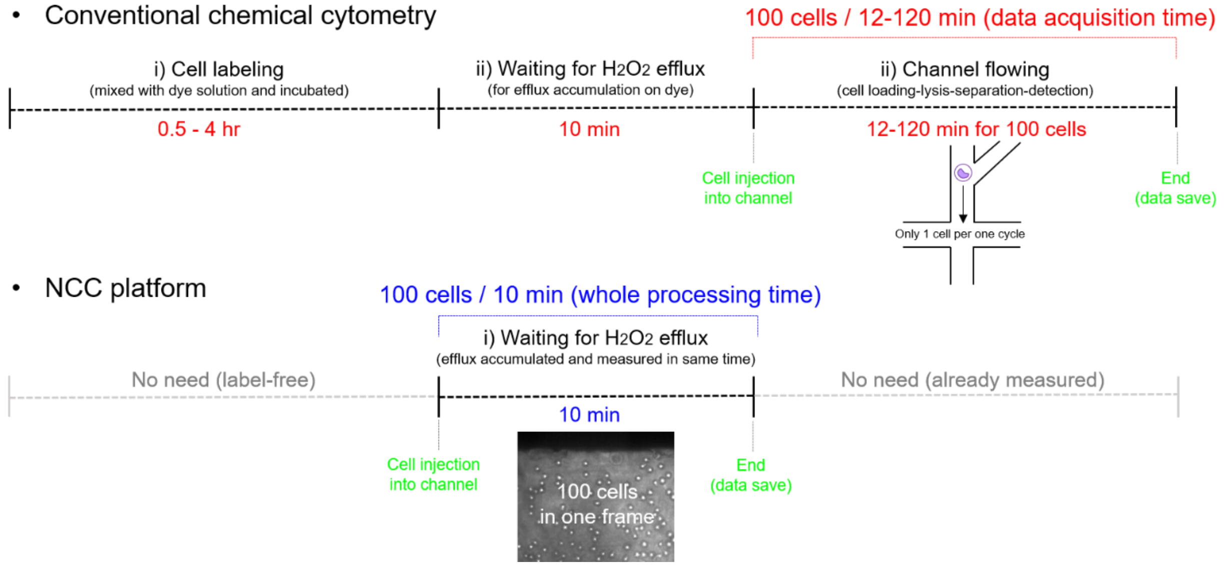

Supplementary Figure 26. Throughput calculations of conventional chemical cytometry and our NCC platform based on the data acquisition time and whole processing time (data acquisition time + efflux detection time (10 min), respectively. Technical throughput of previous image and chemical cytometry fields have been calculated as 'total cell number per data acquisition time' except pre-labeling and incubation time.

10. Parallel channel approach.

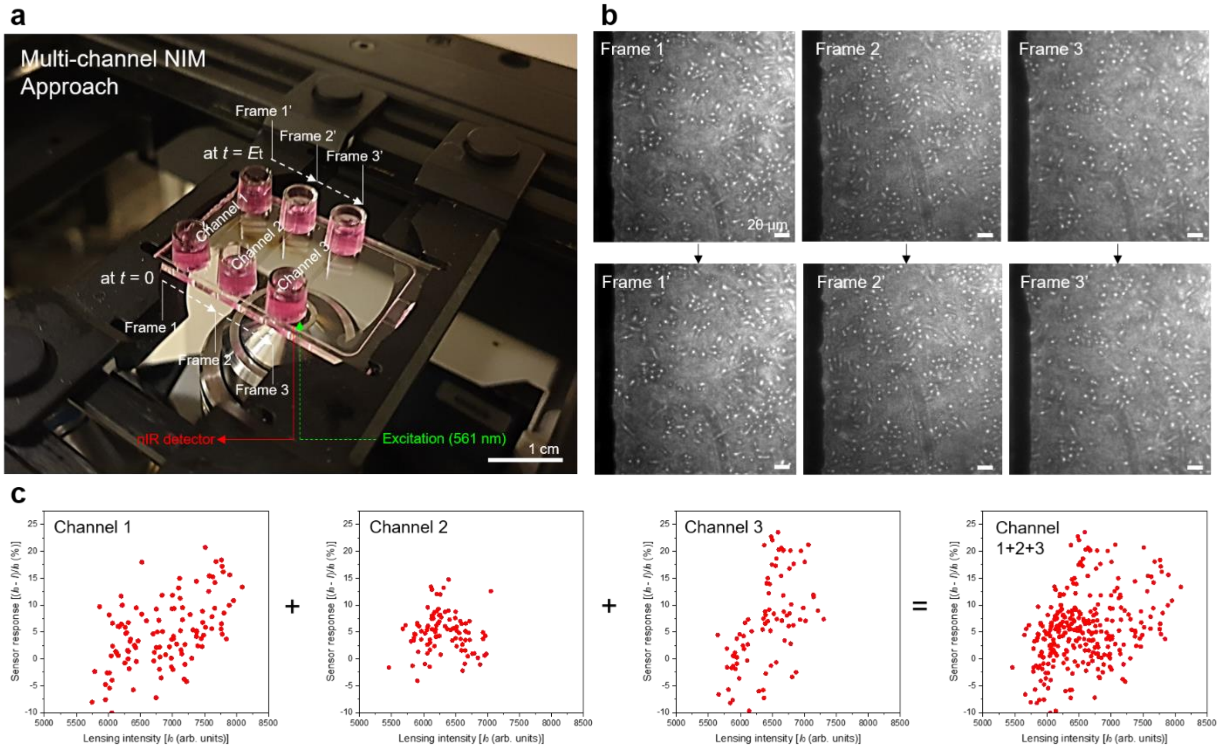

Supplementary Figure 27. Multi-channel NIM approach for large number of cells analyzing. (a) Photograph of parallel three NIM channel within single microfluidic chip. (b) nIR images measured from each single channel at $t = 0$ and E_t. (c) Cytometry plots from each channel and summarized plot of data from each NIM channel.

Since our technique is multiplexable, we can efficiently increase the total analyzing cell number by using multi-channel NIM approach. To demonstrate this, multiple nanosensor channels were fabricated within single microfluidic chip (**Supplementary Figure 27a**). Target cell populations were equally injected through channel 1, channel 2, and channel 3, and fixed spot of each channels were measured at $t = 0$ by stage moving. After waiting efflux time of the cells inside the channel (at $t = E_t$), nIR images of each spots were re-captured by stage moving again (**Supplementary Figure 27b**). Then, nIR sensor response of nanosensor array for E_t could be attracted by frame subtraction of each channel. Accordingly, even though cell number from each flow channel is ~100 cells, total analyzed cell number could be ~300 cells since we used three parallel channel for single microfluidic chip (**Supplementary Figure 27c**).

11. Cell viability assay.

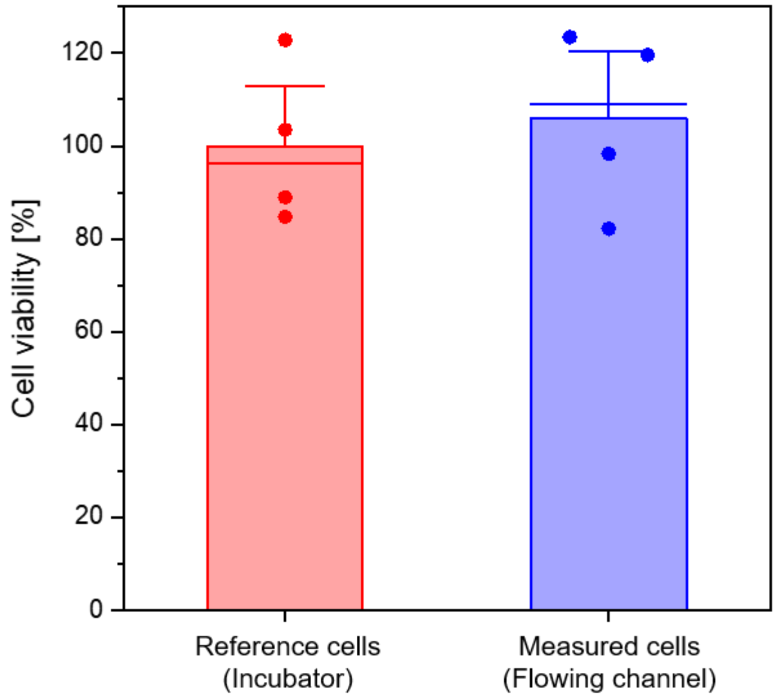

Supplementary Figure 28. Viabilities of cells from incubators (reference) and after flowing channel (measured cell; 10 min excitation laser exposed and flowing inlet-NIM-outlet channel). Data are mean (box) ± σ (error bar), with $n = 4$ technically independent samples.

Viability of the cells were investigated by CellTiter-Glo^{R} 2.0 assay (Promega). Frozen CellTiter-Glo 2.0 reagent was thawed at 4°C overnight and equilibrated to room temperature for 30 min. 200 µL of the media including cells (from incubator for reference and from channel measurement) were loaded on opaque-walled 96-well plates (white for assay; VWR) and 100 µL of reagents were added to each well. Control wells containing media without cells were prepared to determine background luminescence and RPMI-1640 without phenol red (Sigma Aldrich, R1780) was used for the assay. Cells were mixed on an orbital shaker for 10 min to induce cell lysis and incubated at room temperature for 10 min to stabilize the luminescent. Luminescence was measured by microplate reader (Varioskan^{R} Flash, Thermo Scientific) with 485 nm excitation and 520 nm emission reading.

12. Versatility of NCC platform.

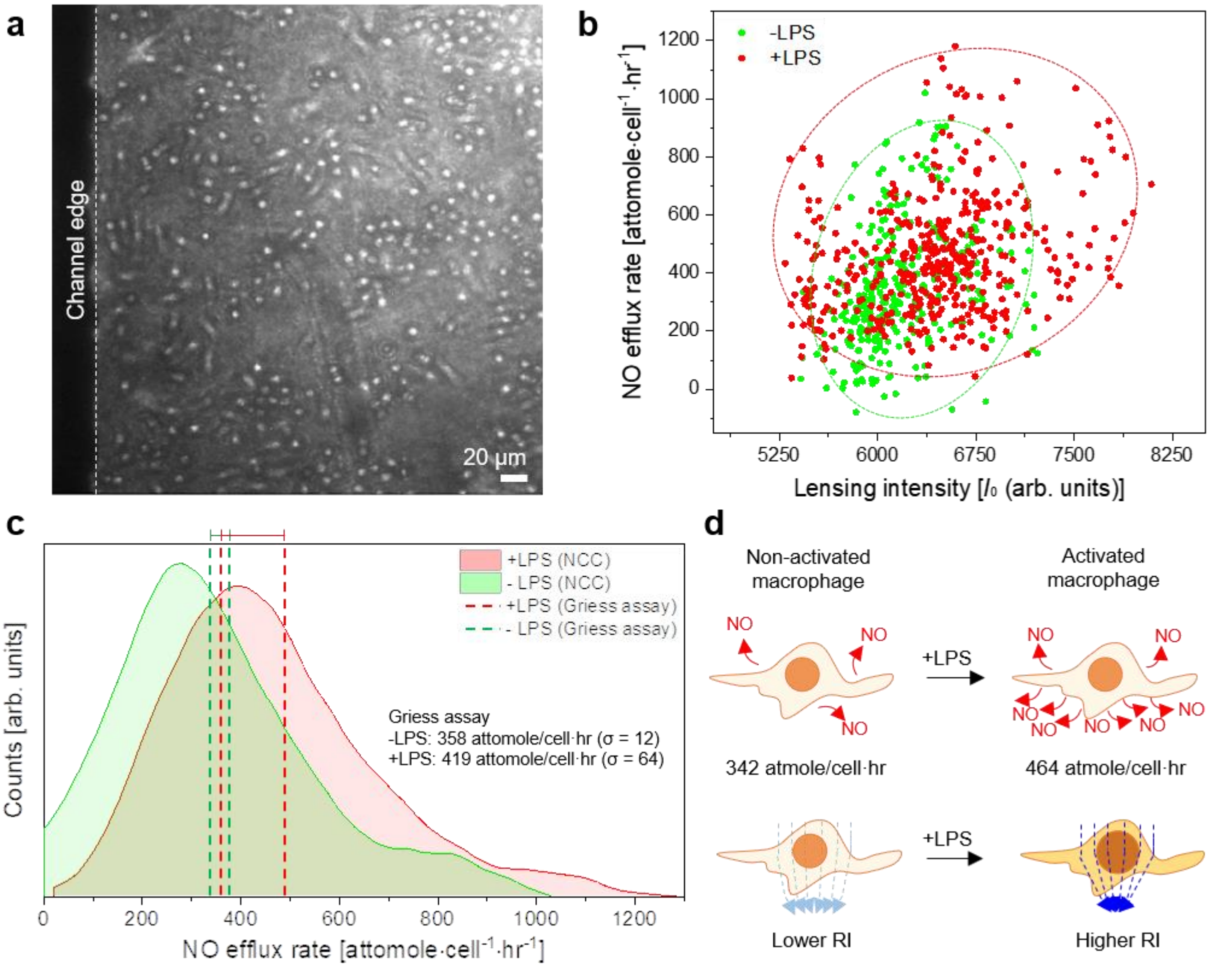

Supplementary Figure 29. iNOS (inducible nitric oxide synthase) heterogeneity monitoring of macrophage populations. (a) nIR image of macrophage in NIM with cell lensing effects. (b) NCC cytometry plots of macrophage populations. Data are n_{cell} = 321 for -LPS, n_{cell} = 405 for +LPS from n = 7 biologically independent samples. (c) NCC distribution curves of NO efflux rates with data from commercial assay kit (Griess assay). (d) Schematics illustrations for cell properties variations of macrophage populations with immune activations.

Additional experiments were conducted to demonstrate the versatility of our technique on completely different cell and efflux: monitoring of inducible nitric oxide synthase (iNOS) heterogeneity of macrophage (Raw 264.7). By quantifying nitric oxide (NO) efflux of single cell, immune mechanism of macrophage and pathogen (*i.e.* lipopolysaccharide (LPS)) can be precisely characterized. New NO nanosensors (SWNT/$(AT)_{15}$) were integrated within

microfluidic channel and target macrophage populations were injection into NIM and let them settled on nanosensor array for efflux measurements. Injected macrophages were densely adhered on nanosensor array with ~200 cells in one frame, showing clear cellular lensing effects (**Supplementary Figure 29a**). To measure NO efflux from iNOS, LPS containing media was added into channel. We plot the real-time NO efflux rates of two distinct groups versus lensing properties of each individual cell (**Supplementary Figure 29b**). Upon LPS immune activation, we find that sensor responses of the single cells from NO efflux increase with increases of I_0, indicating that RI of the cell became higher than non-activated status. This is attributed to that infected macrophage showed higher protein concentration due to the production of iNOS. From these cytometry plots, it is clear that the average NO efflux rate of activated macrophage population was elevated by 35% with a 3% larger increase in the variance of the distribution compared to non-activated populations (**Supplementary Figure 29c**). The nanosensor array allows us to quantify the mean NO efflux rates of these two populations as 342 and 464 attomole/cell·min but with σ of 199 and 206 attomole/cell·min for -LPS and +LPS, respectively. In comparison, we measure average values of 358 (-LPS) and 419 (+LPS) attomole/cell·min from the commercial NO assay kit (Griess method). The mean values are in good agreement for the NCC population and commercial assay. **Supplementary Figure 29d** summarizes the variation in macrophage characteristics before and after the immune activation process that we characterized. Accordingly, we believe that our platform could be widely applied onto unlimited chemical efflux or target cells.
