# Supplementary material for: Cellular lensing and near infrared fluorescent nanosensor arrays to enable chemical efflux cytometry
Source: Nat Commun. 2021 May 25;12:3079. doi: 10.1038/s41467-021-23416-1 (PMC8149711; doi:10.1038/s41467-021-23416-1)
Supplement: Supplementary file 3 — Description of Additional Supplementary Files [file 41467_2021_23416_MOESM3_ESM.pdf]

Description of Additional Supplementary Files

Supplementary Movie 1. Single cell flowing in NIM with nIR lensing effect
