# Supplementary material for: Cellular lensing and near infrared fluorescent nanosensor arrays to enable chemical efflux cytometry
Source: Nat Commun. 2021 May 25;12:3079. doi: 10.1038/s41467-021-23416-1 (PMC8149711; doi:10.1038/s41467-021-23416-1)
Supplement: Supplementary file 5 — Reporting summary [file 41467_2021_23416_MOESM5_ESM.pdf]

nature research

Corresponding author(s): Michael S. Strano

Last updated by author(s): Mar 9, 2021

Reporting Summary

Nature Research wishes to improve the reproducibility of the work that we publish. This form provides structure for consistency and transparency in reporting. For further information on Nature Research policies, see our Editorial Policies and the Editorial Policy Checklist.

Statistics

For all statistical analyses, confirm that the following items are present in the figure legend, table legend, main text, or Methods section.

n/a	Confirmed	
☐	☒	The exact sample size (*n*) for each experimental group/condition, given as a discrete number and unit of measurement
☐	☒	A statement on whether measurements were taken from distinct samples or whether the same sample was measured repeatedly
☐	☒	The statistical test(s) used AND whether they are one- or two-sided<br>*Only common tests should be described solely by name; describe more complex techniques in the Methods section.*
☒	☐	A description of all covariates tested
☐	☒	A description of any assumptions or corrections, such as tests of normality and adjustment for multiple comparisons
☐	☒	A full description of the statistical parameters including central tendency (e.g. means) or other basic estimates (e.g. regression coefficient) AND variation (e.g. standard deviation) or associated estimates of uncertainty (e.g. confidence intervals)
☐	☒	For null hypothesis testing, the test statistic (e.g. *F*, *t*, *r*) with confidence intervals, effect sizes, degrees of freedom and *P* value noted<br>*Give P values as exact values whenever suitable.*
☒	☐	For Bayesian analysis, information on the choice of priors and Markov chain Monte Carlo settings
☒	☐	For hierarchical and complex designs, identification of the appropriate level for tests and full reporting of outcomes
☒	☐	Estimates of effect sizes (e.g. Cohen's *d*, Pearson's *r*), indicating how they were calculated

Our web collection on statistics for biologists contains articles on many of the points above.

Software and code

Policy information about availability of computer code

Data collection	nIR video and images of the cells and microfluidic channel were acquired with PHySpec™ (2.25.3) acquisition software using HDF5 file format.
Data analysis	Statistical tests were performed in OriginPro 2019 and Microsoft Excel 2019. nIR videos and images of the cells, nanosensor array, and microfluidic channel were analyzed using NanoScope Analysis v1.4, ImageJ 1.52p, and MATLAB R2019a.

For manuscripts utilizing custom algorithms or software that are central to the research but not yet described in published literature, software must be made available to editors and reviewers. We strongly encourage code deposition in a community repository (e.g. GitHub). See the Nature Research guidelines for submitting code & software for further information.

Data

Policy information about availability of data

All manuscripts must include a data availability statement. This statement should provide the following information, where applicable:

- Accession codes, unique identifiers, or web links for publicly available datasets
- A list of figures that have associated raw data
- A description of any restrictions on data availability

The authors declare that all data supporting the findings of this study are available within the paper and any raw data can be obtained upon request to the corresponding author.

Field-specific reporting

Please select the one below that is the best fit for your research. If you are not sure, read the appropriate sections before making your selection.

- [x] Life sciences
- [ ] Behavioural & social sciences
- [ ] Ecological, evolutionary & environmental sciences

For a reference copy of the document with all sections, see nature.com/documents/nr-reporting-summary-flat.pdf

Life sciences study design

All studies must disclose on these points even when the disclosure is negative.

Sample size	Sample sizes were chosen based on the availability of the cell culture and microfluidic channel fabrication and the variability of preliminary experiments, with a minimum of n = 3 independent biological replicates to ensure reproducibility of the results. They have been explicitly indicated in the respective table and figure legend.
Data exclusions	No data were excluded from the analysis.
Replication	Reported results were consistently replicated across multiple experiments (minimum of n = 3, each experiments were done in every 2 to 3 days) with all replicates generating similar results.
Randomization	No randomization was necessary for this study because investigators were comparing non-activated and activated cells under well controlled conditions (e.g. variable PMA treatment). No human or animal subjects were used in the study. Randomization is not generally used in this field.
Blinding	Investigators were not blinded. Blinding during collection was not needed because conditions were well controlled. Blinding during analysis was not feasible as the differences between samples under different conditions were apparent in the image. Blinding is also not necessary because the results are quantitative and did not require subjective judgment or interpretation. Blinding is not typically used in the field.

Reporting for specific materials, systems and methods

We require information from authors about some types of materials, experimental systems and methods used in many studies. Here, indicate whether each material, system or method listed is relevant to your study. If you are not sure if a list item applies to your research, read the appropriate section before selecting a response.

Materials & experimental systems

n/a	Involved in the study	
x		Antibodies
	x	Eukaryotic cell lines
x		Palaeontology and archaeology
x		Animals and other organisms
x		Human research participants
x		Clinical data
x		Dual use research of concern

Methods

n/a	Involved in the study	
x		ChIP-seq
x		Flow cytometry
x		MRI-based neuroimaging

Eukaryotic cell lines

Policy information about cell lines

Cell line source(s)	Monocytes (U937, ATCC CRL-1593.2), B lymphocytes (FIB504.64, ATCC HB-293), epithelial (HEK-293, ATCC CRL-1573), and endothelial (HUVEC, ATCC CRL-1730) cells were purchased from American Type Culture Collection (ATCC) and cultivated according to the supplier's protocol.
Authentication	None of the cell lines were authenticated
Mycoplasma contamination	Cell lines were not tested for mycoplasma contamination
Commonly misidentified lines (See ICLAC register)	No commonly misidentified lines were used in the study
